# Phylogenetic distribution of malonate semialdehyde decarboxylase (MSAD) genes among strains within the genus *Mycobacterium*: evidence of MSAD gene loss in the evolution of pathogenic mycobacteria

**DOI:** 10.3389/fmicb.2023.1275616

**Published:** 2023-10-13

**Authors:** Duhyung Lee, Dong Hyun Kim, Hyejun Seo, Seaone Choi, Bum-Joon Kim

**Affiliations:** ^1^Department of Microbiology and Immunology, College of Medicine, Seoul National University, Seoul, Republic of Korea; ^2^Cancer Research Institute, College of Medicine, Seoul National University, Seoul, Republic of Korea; ^3^Department of Biomedical Sciences, College of Medicine, Seoul National University, Seoul, Republic of Korea; ^4^Seoul National University Medical Research Center, Seoul, Republic of Korea; ^5^Brain Korea 21 FOUR Biomedical Science Project, Seoul National University College of Medicine, Seoul, Republic of Korea; ^6^Liver Research Institute, College of Medicine, Seoul National University, Seoul, Republic of Korea

**Keywords:** malonate semialdehyde decarboxylase (MSAD), *Mycobacterium*, phylogenetic distribution, MSAD-1, MSAD-2, *Mycobacterium avium* subsp. *paratuberculosis*

## Abstract

Despite the great diversity of malonate semialdehyde decarboxylases (MSADs), one of five subgroups of the tautomerase superfamily (TSF) found throughout the biosphere, their distribution among strains within the genus *Mycobacterium* remains unknown. In this study, we sought to investigate the phylogenetic distribution of MSAD genes of mycobacterial species via genome analysis of 192 different reference *Mycobacterium* species or subspecies retrieved from NCBI databases. We found that in a total of 87 of 192 strains (45.3%), MSAD-1 and MSAD-2 were distributed in an exclusive manner among *Mycobacterium* species except for 12 strains, including *Mycobacterium chelonae* members, with both in their genome. Of note, *Mycobacterium* strains better adapted to the host and of high virulence potential, such as the *Mycobacterium tuberculosis* complex, *Mycobacterium leprae*, *Mycobacterium marinum*, *Mycobacterium ulcerans*, and *Mycobacterium avium* subsp. *paratuberculosis*, had no orthologs of MSAD in their genome, suggesting MSAD loss during species differentiation in pathogenic slow-growing *Mycobacterium*. To investigate the MSAD distribution among strains of *M. avium* subspecies, the genome sequences of a total of 255 reference strains from the four subspecies of *M. avium* (43 of subspecies *avium*, 162 of subspecies *hominissuis*, 49 of subspecies *paratuberculosis*, and 1 of subspecies *silvaticum*) were further analyzed. We found that only 121 of 255 strains (47.4%) had MSADs in their genome, with none of the 49 *M. avium* subsp. *paratuberculosis* strains having MSAD genes. Even in 13 of 121 *M. avium* strains with the MSAD-1 gene in their genome, deletion mutations in the 98th codon causing premature termination of MSAD were found, further highlighting the occurrence of MSAD pseudogenization during species or subspecies differentiation of *M. avium.* In conclusion, our data indicated that there are two distinct types of MSADs, MSAD-1 and MSAD-2, among strains in the *Mycobacterium* genus, but more than half of the strains, including pathogenic mycobacteria, *M. tuberculosis* and *M. leprae*, have no orthologs in their genome, suggesting MSAD loss during host adaptation of pathogenic mycobacteria. In the future, the role of two distinct MSADs, MSAD-1 and MSAD-2, in mycobacterial pathogenesis or evolution should be investigated.

## 1. Introduction

The genus *Mycobacterium* is composed of more than 200 established and validated species and subspecies belonging to the phylum *Actinobacteria*, defined by a rod-shaped morphology, acid fastness, unusual cell walls containing mycolic acids, and relatively high genomic DNA G+C contents (∼ 61 to 71%) (Vincent Lévy-Frébault and Portaels, 1992; Jankute et al., 2015; Gupta et al., 2018). Generally, *Mycobacterium* can be separated into two groups according to their pathogenic potential: human pathogens, including *Mycobacterium tuberculosis* and *Mycobacterium leprae*, which cause tuberculosis and leprosy, respectively, and nontuberculous *Mycobacterium* (NTM), which are environmental mycobacteria that do not cause tuberculosis, as the name suggests, and are often nonpathogenic to humans and animals (Gagneux, 2018; Johansen et al., 2020; van Hooij and Geluk, 2021). The genus can be further separated into two groups, slow-growing *Mycobacterium* (SGM) (i.e., requiring more than 7 days to form visible colonies on solid agar) and rapid-growing *Mycobacterium* (RGM) requiring <7 days to form colonies (Lotti and Hautmann, 1993). Although most NTMs are found in the environment, such as soil or natural and drinking water sources, a few species, including the *Mycobacterium avium complex* (MAC) and *Mycobacterium abscessus*, often cause serious lung diseases through infections in humans (Nishiuchi et al., 2017; Johansen et al., 2020; Ratnatunga et al., 2020). The increases in immunosuppressive drug use, broad-spectrum antibiotic therapy, and patients with underlying lung diseases, including cystic fibrosis and bronchiectasis, have contributed to the recent rise in the global incidence of NTM infections in developed countries (Petrini, 2006; Chalmers et al., 2018; To et al., 2020).

The tautomerase superfamily (TSF) consists of more than 11,000 members throughout the biosphere, which can be classified into five major categories: 4-oxalocrotonate tautomerase (4-OT), 5-(carboxymethyl)-2-hydroxymuconate isomerase (CHMI), macrophage migration inhibitory factor (MIF), *cis*- and *trans*-3-chloroacrylic acid dehalogenase (*cis*-CaaD and CaaD, respectively), and malonate semialdehyde decarboxylase (MSAD) (Poelarends et al., 2008a; Davidson et al., 2018). Despite some exceptions, they share a β-α-β structure with an unusual catalytic amino-terminal proline as a general base. MSAD not only catalyzes the decarboxylation of malonate semialdehyde to produce acetaldehyde but also participates in degradative pathways of 1,3-dichloropropene, a soil fumigant (Poelarends and Whitman, 2004).

To date, structural studies regarding MSAD have been mainly focused on nine organisms, originating from various species such as *Pseudomonas pavonaceae* 170 (PpMSAD) (Poelarends et al., 2003, 2004, 2005; Almrud et al., 2005), *Coryneform bacterium* strain fg41 (FG41 MSAD) (Poelarends et al., 2008b; Guo et al., 2013), *Lactobacillus casei* strain BL23 (IolK) (Poelarends et al., 2008b), *Bacillus subtilis* strain 168 (YusQ, YodA, and YrdN) (Poelarends et al., 2005), *Burkholderia phymatum* strain STM815 (Bp4401) (Huddleston et al., 2014), *Calothrix* sp. PCC 6303(437) (Lancaster et al., 2022), and *Rivularia* sp. PCC 7116(JJ3) (Lancaster et al., 2022). However, research on the MSADs of *Mycobacterium* strains is limited.

Comparative genomic studies have revealed that overt human-pathogenic *Mycobacterium* species, including *M. tuberculosis, M. leprae*, and *Mycobacterium ulcerans*, have undergone genome reduction and gene loss since their evolution from the ancestor (Gutierrez et al., 2005; Marri et al., 2006; Gómez-Valero et al., 2007; Qi et al., 2009). Since MSAD plays a key role in bacterial metabolism, it may have had distinct effects on the evolutionary scenario of *Mycobacterium* species in terms of their groups, pathogenic or environmental strains and slow-growing or rapid-growing status. Therefore, investigation of the phylogenetic distribution of MSAD genes among *Mycobacterium* strains would provide novel insight into their evolution and pathogenesis.

In the present study, we sought to investigate the phylogenetic distribution of MSAD genes of mycobacterial species via genome analysis of 192 different reference *Mycobacterium* species or subspecies retrieved from NCBI databases. In addition, to further test our hypothesis of MSAD loss in *Mycobacterium* strains more adapted to host-associated life, we further checked the distribution of MSADs among 255 MAC strains of the four subspecies of *M. avium* retrieved from NCBI databases.

## 2. Materials and methods

### 2.1. *Mycobacterium* type strain database

**FIGURE 1:**
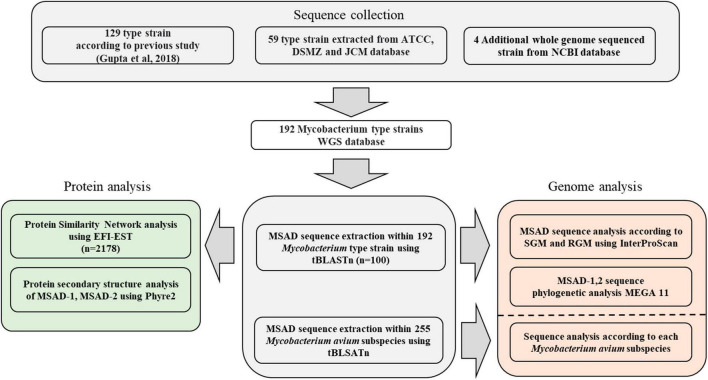
Workflow representing the construction of the *Mycobacterium* genome sequence database and software used for each analysis in this study.

A total of 192 type strains of *Mycobacterium* with whole genome sequences present in the NCBI taxonomy browser, ATCC (American Type Culture Collection), DSMZ (German Collection of Microorganisms and Cell Cultures), and JCM (Japan Collection of Microorganisms) were selected (Figure 1). Of these, 129 type strains were referenced from a previous study (Gupta et al., 2018), and the other 67 type strains were selected from the ATCC (21 strains), DSMZ (34 strains), and JCM (4 strains). In addition, we selected four newly identified *Mycobacterium* species, *Mycobacterium dioxanotrophicus*, *Mycolicibacterium nivoides*, *Mycobacterium terramassiliense*, and *Mycobacterium senriense* (He et al., 2017; Bouam et al., 2018; Dahl et al., 2021; Abe et al., 2022), with their whole genome sequences registered with the NCBI and added them to the database. The whole-genome sequencing project names and accession numbers are listed in Supplementary Table 1.

### 2.2. MSAD sequence data collection

Using two MSAD sequences (MSAD-1: NCBI accession number OLT81431.1 and MSAD-2: OLT83119.1) of *Mycobacterium chelonae* subsp. *chelonae* ATCC 35752^T^ as query sequences, the presence of MSAD proteins was confirmed in 192 *Mycobacterium* type strains through the tBLASTn algorithm (Supplementary Table 1; Altschul et al., 1990, 1997). Among the BLAST search results, sequences were extracted based on a cutoff of 90% for query coverage and 10^–20^ for the *E*-value. MSAD sequences ranging in length from 119 to 137 amino acids were collected. A total of 100 MSAD sequences from 192 *Mycobacterium* type strains were extracted. The extracted protein sequences of MSAD orthologs are stored in FASTA format (Supplementary Data 1) and their DNA sequences are listed in Supplementary Table 2.

### 2.3. Protein similarity network of the total MSAD family

A total of 2,078 MSAD sequences, which are widely distributed throughout the biosphere, are registered in the SFLD (Superfamily of Ligand-Binding Protein Database) (Akiva et al., 2014; Davidson et al., 2018). They were trimmed and aligned before being submitted to the Enzyme Function Initiative enzyme similarity tool (EFI-EST) webserver for analysis (Zallot et al., 2019) (Job ID 96068). The EFI-EST was used to create the sequence similarity network (Oberg et al., 2023). We aligned total MSAD protein sequences (2,078 sequences) and mycobacterial MSADs (100 sequences) together and submitted them to the EFI-EST algorithm. Merged MSAD sequences were stored in FASTA format (Supplementary Data 2). In this study, the *E*-value for SSN Edge calculation was set to 5, and the convergence ratio was set to 0.743. This value decreases from 1.0 for sequences that are very similar (identical) to 0.0 for sequences that are very different (unrelated). Constructed networks were then transferred to the Color SSN utility for Representative Node (RepNode) Networks (Job ID 96072). The 100% identity RepNode network was stored as Supplementary Data 3, and 50% identical sequences were grouped to reduce the total number of nodes. The colored SSN was visualized in Cytoscape (Shannon et al., 2003) (version 3.9.1).

### 2.4. Protein structure prediction of MSAD

Four MSAD sequences of *M. avium* (MSAD-1 of slow growing mycobacteria), *M. abscessus* (MSAD-1 of rapid growing mycobacteria), *Mycobacterium terrae* (MSAD-2 of slow growing mycobacteria), and *Mycobacterium fortuitum* (MSAD-2 of rapid growing mycobacteria) were submitted to the phyre2 webserver^1^ for protein function prediction (Kelley et al., 2015). The structures of four stains of MSAD (two strains of MSAD-1 and two strains of MSAD-2) were determined based on their protein sequences (Supplementary Data 1). The constructed MSAD 3D models were then compared to PDB-registered proteins. The protein derived from the *C. bacterium* strain fg41 registered in the PDB (PDB ID: 3MJZ) was used as a template to align MSAD sequence of *M. abscessus* and *M. terrae*. And the PpMSAD derived from the *P. pavonaceae* registered in the PDB (PDB ID: 2AAL) was used as a template to align MSAD sequence of *M. avium* and *M. fortuitum*. The protein structures created with phyre were analyzed with the PyMOL Molecular Graphics System Schrödinger, Inc. (version 2.5.5). The identified *Mycobacterium* type strains were used to predict the structure and function of MSAD sequences using InterProScan (Jones et al., 2014).

### 2.5. MSAD sequence alignment and phylogenetic analysis

A total of 57 MSAD-1 sequences and 43 MSAD-2 sequences from *Mycobacterium* reference strains were aligned using the MUSCLE method for each DNA sequence and amino acid sequence through the MEGA 11 program (Tamura et al., 2021). The DNA and protein sequence-based phylogenetic trees of MSAD-1 and MSAD-2 were constructed through the maximum-likelihood method. Branch support value was calculated through 100 bootstrap replications. Phylogenetic trees based on 644 bp *hsp65* sequences are often used to classify and identify *Mycobacterium* species (Kim et al., 2005). The *hsp65* sequence of *M. tuberculosis* H37Rv (GenBank accession number M15467) was used to extract the *hsp65* sequence from whole genome sequences of the type strains through BLAST (Supplementary Data 4). In this study, two *hsp65* sequence-based trees (one for MSAD-1 sequences from 56 mycobacterial strains and the other for MSAD-2 sequences from 43 strains) were constructed through MUSCLE alignment and the maximum-likelihood method. Branch support value was calculated through 100 bootstrap replications. YrdN (MSAD of *B. subtilis* strain 168) was used as an outgroup in the MSAD tree, while *Tsukamurella paurometabola* KCTC 9821^T^ (GenBank accession number UHIQ01000001.1) was used as an outgroup in the *hsp65* tree.

### 2.6. *Mycobacterium avium* subspecies strain MSAD sequence analysis

To analyze MSAD protein retention within *M. avium* subspecies, a total of 255 whole genome sequences were obtained from the NCBI database, including 43 *M. avium* subsp. *avium* sequences, 162 *M. avium* subsp. *hominissuis* sequences, 49 *M. avium* subsp. *paratuberculosis* sequences, and 1 *M. avium* subsp. *silvaticum* sequence. In total, 121 MSAD sequences were extracted from the whole genomes of *M. avium* subspecies using tBLASTn (Altschul et al., 1997). The MSAD sequences were aligned and analyzed using the MUSCLE method through MEGA 11 (Tamura et al., 2021). Information regarding the accession numbers, metadata, and all publicly available assemblies for the whole genome sequences was also extracted from the NCBI database (Supplementary Data 5). Extracted MSAD sequences were stored in FASTA format (Supplementary Data 6).

### 2.7. Preparation of *Mycobacterium abscessus* MSAD-1 protein

Recombinant MSAD-1 protein of *M. abscessus* (NCBI accession number OLT57519.1) were purified from *Escherichia coli* as previously described with minor modification (Jeong et al., 2022). Briefly, the DNA sequence of MSAD-1 was amplified from *M. abscessus* ATCC 19977^T^ using PCR with following primer sets (forward primer, 5′-TTT GGA TCC ATG CCA TTG GTG CGC ATC GAC CTC-3′; reverse primer, 5′-AAA AAG CTT GTG CGC CTG CGG CGG GCA C-3′), and cloned into pET-28a. The expression and purification of MSAD-1 were commercially commissioned by Bionics (Seoul, Republic of Korea). In detail, the protein expression was induced in *E. coli* Rosetta2 (DE3) strains (Novagen, WI, USA) transformed with pET28a-MSAD-1 by adding 1 mM isopropyl β-D-thiogalactopyranoside (IPTG) at 26°C for 6 h. Cultured bacterial cells were harvested and sonicated for 30 cycles at 70% amplitude. After centrifuge, the supernatant was purified with HisTrap™ HP His tag protein purification columns (Cytiva, MA, USA) for Ni-NTA affinity chromatography via ÄKTA go system (Cytiva, MA, USA). Purified proteins were subjected to endotoxin removal using Pierce™ high-capacity endotoxin removal spin columns (Thermo Scientific, MA, USA) and quantified by Pierce™ chromogenic endotoxin quant kit (Thermo Scientific, MA, USA).

### 2.8. Induction of pro-inflammatory cytokines, TNF-α and IL-6 on J774A.1 cells by MSAD-1

The murine macrophage cell line, J774A.1, was maintained at 37°C with 5% CO_2_ in RPMI 1640 supplemented with 10% (v/v) fetal bovine serum (FBS) and 1% penicillin-streptomycin (PS). To induce protein uptake efficiently, J774A.1 cells were starved in reduced serum medium, opti-MEM (Gibco, MT, USA), at 37°C for 1 h. Subsequently, they were incubated with various concentrations of MSAD-1 or 100 ng/ml LPS (Sigma, MO, USA) in RPMI 1640 supplemented with 2% (v/v) FBS and 1% PS at 37°C for either 24 or 48 h. The culture medium was used to measure the levels of the cytokines using an enzyme-linked immunosorbent assay (ELISA) kit (Invitrogen, MA, USA) according to the manufacture’s instructions.

## 3. Results

### 3.1. Construction of a novel MSAD protein similarity network including 100 mycobacterial MSAD sequences

**FIGURE 2:**
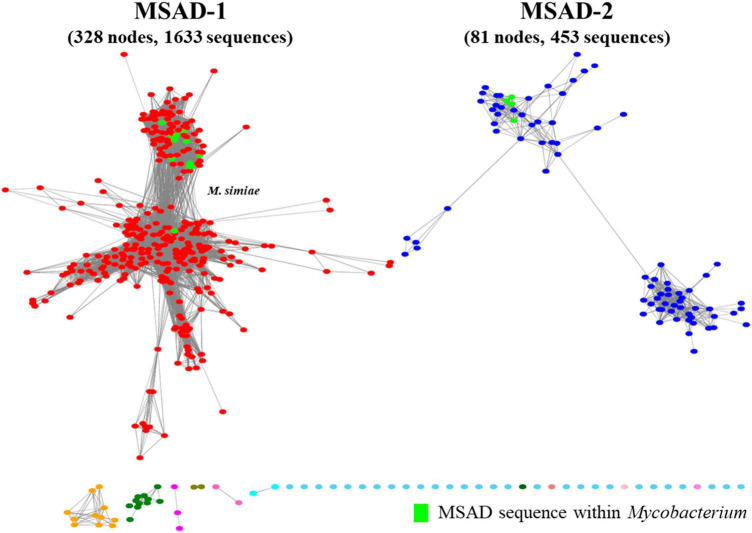
A new malonate semialdehyde decarboxylase (MSAD) protein similarity network including mycobacterial MSADs constructed in this study. Visualized protein similarity network of MSADs within the biosphere. Total MSAD family protein sequences (2,078 sequences) and mycobacterial MSADs (100 sequences) were used to construct the network and the network was visualized through the EFI-EST webserver. MSADs consist of two independent branches, MSAD-1 (red) and MSAD-2 (blue). The MSAD-1 branch consists of 328 nodes with 1,633 sequences. The MSAD-2 branch consists of 81 nodes with 453 sequences. All mycobacterial MSADs were scattered among the MSAD-1 and MSAD-2 groups.

To examine the distribution of a total of 100 mycobacterial MSAD orthologs extracted from genome sequences of 192 strains in this study among all the biospheres, we constructed a novel protein similarity network including a total of 2,178 MSAD protein sequences from established MSAD protein sequences (2,078 sequences) (Akiva et al., 2014) and mycobacterial MSADs (100 sequences) extracted in this study and submitted to the EFI-EST algorithm (Zallot et al., 2019; Figure 1). All 2,178 MSADs are grouped into 12 clusters of 472 nodes, which are connected by 7,677 edges, and further clustered into two major groups (colored red and blue) (Figure 2). While one group (colored red) consists of 328 larger nodes of 1,633 sequences, including PpMSAD (Poelarends et al., 2003) and FG41 MSAD (Poelarends et al., 2008b), which have been widely studied for the elucidation of MSAD function, the other group consists of 81 smaller nodes of 453 MSAD sequences. We decided to designate these two groups as MSAD-1 and MSAD-2, respectively. Generally, all 100 mycobacterial MSAD sequences extracted in this study also belonged to these two major groups, MSAD-1 and MSAD-2 (Figure 2). However, there are some discrepancies in their distribution in the protein similarity network. Strains of MSAD-1 are more widely scattered among 13 nodes compared with those of MSAD-2, found at 4 nodes, suggesting more sequence divergence between MSAD-1 strains than between MSAD-2 strains (Figure 2).

### 3.2. The distribution of MSAD genes among *Mycobacterium* strains

**FIGURE 3:**
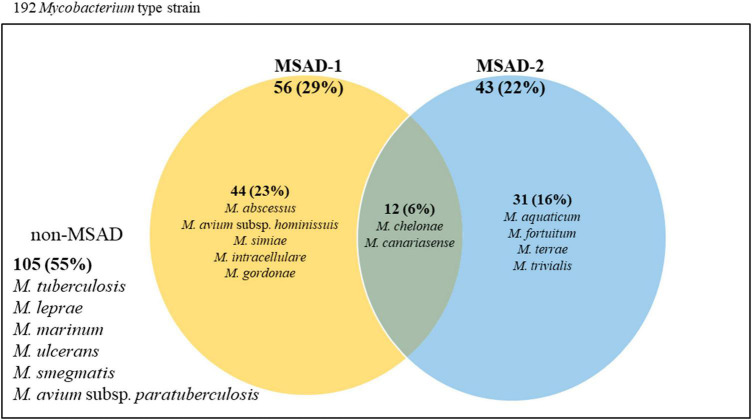
Venn diagram representing the distributions of MSAD-1 and MSAD-2 among strains within the genus *Mycobacterium.* Of all 192 *Mycobacterium* reference strains, only 87 (45.3%) strains have MSAD genes in their genome. Of these, 74 strains have either MSAD-1 or MSAD-2 genes in their genome, and 12 strains, including three from subspecies of *M. chelonae*, have both MSAD-1 and MSAD-2 genes. The remaining 105 strains, including strains of *M. tuberculosis* and *M. leprae*, do not have MSAD-like genes.

Of 192 *Mycobacterium* reference strains, only fewer than half (87, 45.3%) had MSAD genes in their genomes (105 strains without any MSAD orthologs in their genomes) (Figure 3). The DNA sequences and accession numbers of all 100 MSADs from 87 *Mycobacterium* strains are presented in Supplementary Table 1. Of all 100 MSAD sequences, 57 and 43 belonged to MSAD-1 and MSAD-2, respectively. Most *Mycobacterium* strains (75 strains) have a single MSAD in their genomes, MSAD-1 or MSAD-2, in an exclusive manner (74 strains have single copy of MSAD gene and *Mycobacterium simiae* with two copies of MSAD-1). The remaining 12 species, including the three subspecies *M. chelonae* subsp. *chelonae*, *M. chelonae* subsp. *bovis* (Kim et al., 2017), and *M. chelonae* subsp. *gwanakae* (Kim et al., 2018), have both types of MSADs, MSAD-1 and MSAD-2, in their genomes (Supplementary Table 1). Of note, overt slow-grower human pathogens, including *M. tuberculosis, M. leprae*, *Mycobacterium marinum, M. ulcerans*, and *M. avium* subsp. *paratuberculosis*, do not have any MSAD genes in their genomes (Supplementary Table 1 and Figure 3). In addition, of 49 slow-growing strains with an MSAD gene, all of those from the Tuberculosis-Simiae clade (emended genus *Mycobacterium*) have the MSAD-1 type but not the MSAD-2 type in their genomes. However, all members of the Terrae clade (*Mycolicibacter* gen. nov.) and Triviale clade (*Mycolicibacillus* gen. nov.) have MSAD-2 but not MSAD-1. Our finding of no MSAD genes in the genomes of more than half the *Mycobacterium* species, particularly in overt human pathogens, suggests gene loss during the evolutionary adaptation of *Mycobacterium* towards host-associated lifestyles, particularly in slow-growing human pathogens. In addition, the finding that two slow grower groups, the Tuberculosis-Simiae clade and the *M. terrae* complex group, including the Terrae and Triviale clades, have distinct MSAD types, MSAD-1 and MSAD-2, respectively (Supplementary Table 1 and Figure 3), suggests the distinct roles of the two MSAD types in slow-grower evolution.

### 3.3. Distinct primary structures between MSAD-1s of slow-grower, MSAD-1s of rapid-grower and MSAD-2s

**FIGURE 4:**
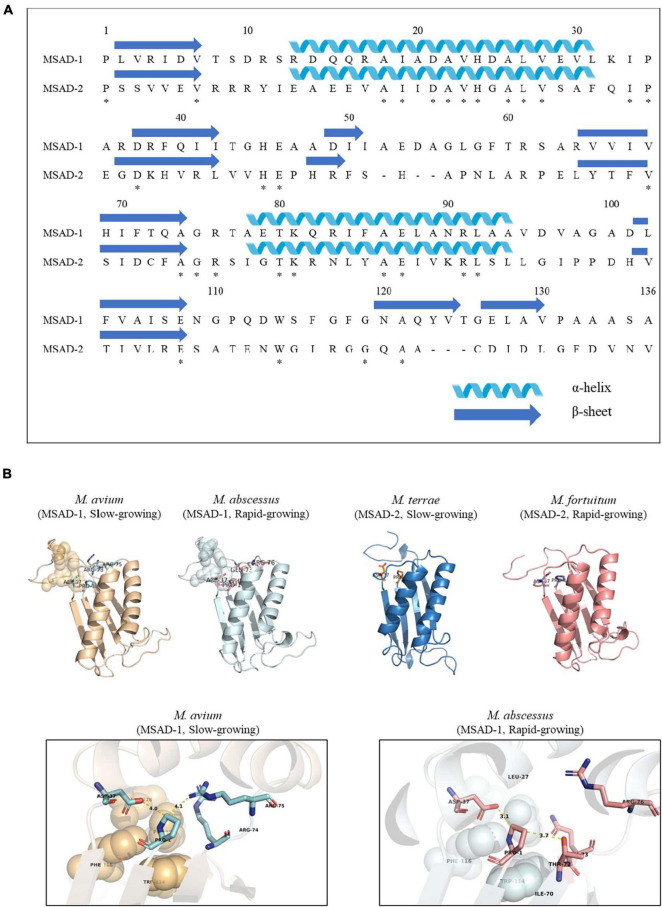
The primary and tertiary structures of mycobacterial MSAD-1 and MSAD-2 proteins. This number corresponds to the codon number of the *P. pavonaceae* MSAD protein. **(A)** Primary structure of MSAD-1 and MSAD-2 of *M. chelonae* ATCC 35752^T^ (MSAD-1: NCBI accession number OLT81431.1 and MSAD-2: OLT83119.1). The arrow indicates the β-sheet structure, while the spiral indicates the α-helix structure. **(B)** Ribbon diagram representing predicted tertiary structures of MSAD-1 of *M. avium* (slow-grower, left), MSAD-1 of *M. abscessus* (rapid-grower, center left), MSAD-2 of *M. terrae* (slow-grower, center right), and MSAD-2 of *M. fortuitum* (rapid-grower, right). MSAD-1 types showed a hydrophobic wall (shown as sphere) behind the active site (shown as stick) of Pro-1 and Asp-37. The structure of MSAD-2 is the same as MSAD-1 in that the positions of Pro-1 and Asp-37 located. *Identical amino acids between two MSADs.

Our protein similarity network analysis indicated that all 100 mycobacterial MSADs consisted of a total of 17 nodes, with 13 nodes for MSAD-1s and 4 nodes for MSAD-2s (Figure 2). The predicted structures of both MSAD types of *M. chelonae* were identified as consistent with the MSAD model (PDB FG41) with 100% confidence, despite low protein percent identity between strains of MSAD-1 and MSAD-2 (75 and 25%, respectively). Both MSAD-1s and MSAD-2s share the characteristic structures of MSADs such as the β-α-β structure and proline-1 sequences of the TSF signature (Figure 4A; Davidson et al., 2018).

Tertiary structure prediction was performed for several *Mycobacterium* species according to each type. One of the template MSADs originating from *P. pavonaceae* (PpMSAD) have critical amino acids that facilitate binding to substrates (Poelarends et al., 2003, 2004, 2005; Almrud et al., 2005). Briefly, the Pro-1 and β-α-β structure shape is required for MSAD enzyme activity. Asp-37 and a pair of arginines, Arg-73 and Arg-75, are thought to act as linkers in the enzyme–substrate complex. Trp-114, Phe-116, Phe-123, and Leu-128 also participate in the stabilization of the substrate by forming a hydrophobic wall in the MSAD homotrimer (Almrud et al., 2005). Meanwhile, one of the other template MSAD originated from *C. bacterium* strain fg41 (FG41 MSAD) has a distinct primary structure compared with that of PpMSAD (Poelarends et al., 2008b; Guo et al., 2013). Despite also having Pro-1, a β-α-β structure shape, and Asp-37, similar to PpMSAD, FG41 MSAD has different signature protein sequences, indicating that the mechanisms of the two MSADs are different. Side chains of Thr-72, Gln-73, Arg-76, and Tyr-123 replace the pair of arginine residues in FG41 MSAD (Figure 4B).

**FIGURE 5:**
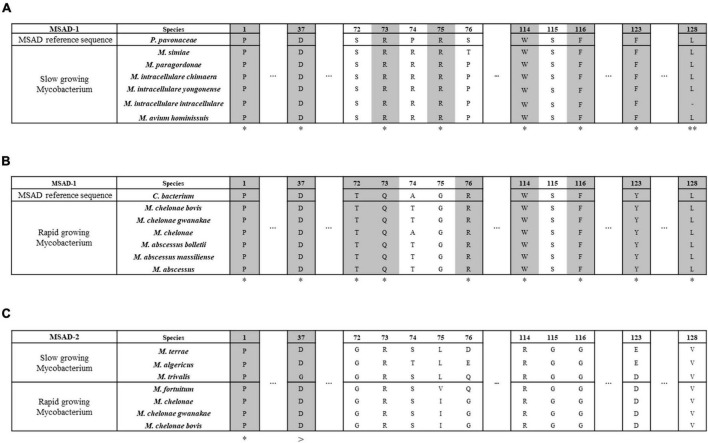
Distinct primary structures between mycobacterial MSAD-1 of slow-growers and rapid-growers and MSAD-2 of slow-growers and rapid-growers. This number corresponds to the codon number of the *P. pavonaceae* MSAD protein (PpMSAD). **(A)** The primary structure of MSAD-1 of slow-growers. MSAD-1 of slow-growers has structural characteristics similar to those of *P. pavonaceae* MSAD (PpMSAD), such as a pair of arginine (Arg-73 and Arg-75) residues and a hydrophobic wall (Trp-114, Phe-116, Phe-123, and Leu-128). **(B)** The primary structure of MSAD-1 of rapid-growers. MSAD-1 of rapid-growers has structural characteristics similar to those of *Coryneform bacterium* MSAD (FG41 MSAD), such as side chains (Thr-72, Gln-73, and Tyr-123) and a hydrophobic wall (Trp-114, Phe-116, and Leu-128). **(C)** The primary structure of MSAD-2. MSAD-2 does not have any specific sequence related to the enzymatic reaction of MSAD, regardless of the growth characteristics of *Mycobacterium*. *Indicates that all type strains have the same nucleotide. **Means that most of them have the same nucleotide. >Is the same nucleotide except for the trivalis clade.

Our data showed that all 57 mycobacterial MSAD-1 proteins have Pro-1, Asp-37, Trp-114, Phe-116, and Leu-128 (Figure 5). However, there are differences between rapid-growers and slow-growers in the substrate interaction region. While the MSAD-1 of rapid-growers has Thr-72 and Gln-73 except in *Mycobacterium aubagnense* and *Mycobacterium phocaicum*, the MSAD-1s of slow-growers have the Arg-73 and Arg-75 pair without exception, similar to PpMSAD (Figures 5A, B).

We also found that mycobacterial MSAD-2 has a distinct primary structure compared with that of MSAD-1. All MSAD-2 groups also start with Pro-1, and most of them have Asp-37 (Figure 5C). However, the MSAD-2 of *Mycobacterium koreense*, *Mycobacterium parakoreense*, and *Mycobacterium trivialis* belonging to the Triviale clade encodes Asn-37 instead of Asp-37. Moreover, unlike MSAD-1, MSAD-2 has no other signature sequences, including the pair of arginines (Arg-73 and Arg-75) or sequences related to the hydrophobic wall (Figure 5C). Our data showed that sequences of MSAD-2 are more conserved between strains (61.6–100%) than those of the MSAD-1 group (29.7–100%) (Supplementary Figure 1A). Together, our data indicated that there are three distinct primary protein structures of MSADs between *Mycobacterium* strains (MSAD-1 of slow-growers, MSAD-1 of rapid-growers, and MSAD-2), suggesting their distinct roles in the evolution and pathogenesis of *Mycobacterium*.

### 3.4. Phylogenetic analysis based on MSAD-1 and MSAD-2 sequences

**FIGURE 6:**
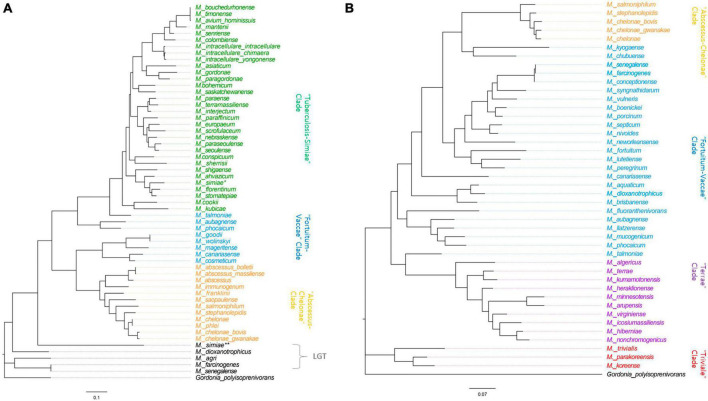
Phylogenetic trees based on the MSAD-1 (348–411 bp) and MSAD-2 (357–435 bp) sequences. **(A)** The mycobacterial MSAD-1 phylogenetic tree was constructed from DNA sequences of 57 mycobacterial MSAD-1s from 56 mycobacterial strains (two independent MSAD-1s in *M. simiae*) with DNA lengths ranging from 348 to 411 bp. **(B)** The MSAD-2 phylogenetic analysis was constructed using 43 DNA sequences of mycobacterial MSAD-2s, with DNA sequence lengths ranging from 357 to 435 bp.

To assess the phylogenetic relationships of MSAD-1s and MSAD-2s among *Mycobacterium* strains, we performed phylogenetic analysis based on mycobacterial MSAD-1 and MSAD-2 sequences. First, the mycobacterial MSAD-1 phylogenetic tree was constructed from DNA sequences of 57 mycobacterial MSAD-1s from 56 *Mycobacterium* strains (two independent MSAD-1s in *M. simiae*) with DNA lengths ranging from 348 to 411 bp. The G+C content of MSAD-1 ranges from 56.1 to 68.4%. We found that most *Mycobacterium* strains can be separated at the species level, showing sequence similarity levels ranging from 29.7 to 100% (Supplementary Figure 1A). In general, the MSAD-1 DNA sequence-based phylogenetic tree revealed natural relationships between *Mycobacterium*, as shown in the *hsp65*-based tree (Supplementary Figure 2), clearly including separation between slow-growers (Tuberculosis-Simiae clade) and rapid-growers and separation between the Fortuitum-Vaccae clade and Abscessus-Chelonae clade. Of note, our MSAD-1-based phylogenetic analysis showed that the MSAD of five strains (*M. simiae, M. dioxanotrophicus, Mycobacterium farcinogenes, Mycobacterium senegalense*, and *Mycobacterium agri*) did not belong to the *Mycobacterium* clade, suggesting that their MSAD-1s was laterally transferred from another bacterial group (Figure 6A). In parallel, our further BLASTn analysis also supports LGT transfer of their MSAD-1 genes (Supplementary Table 3). Phylogenetic analysis based on MSAD-1 protein sequences also showed a topology similar to that based on MSAD-1 DNA sequences (Supplementary Figure 3).

MSAD-2 phylogenetic analysis was performed using 43 DNA sequences of mycobacterial MSAD-2, with DNA sequence lengths ranging from 357 to 435 bp. The G+C content of MSAD-2 is slightly higher than that of MSAD-1, ranging from 58 to 69.5%, and most strains can be separated at the species level, showing sequence similarity levels ranging from 61.6 to 100%, indicating that MSAD-2 is more conserved than MSAD-1 (Supplementary Figure 1B). Our MSAD-2 phylogenetic analysis also reveals natural relationships between mycobacterial strains, including separation between four clades, the Fortuitum-Vaccae and Abscessus-Chelonae clades of rapid-growers and Terrae and Triviale clades of slow-growers (Figure 6B), which is also shown in the MSAD-2 protein-based phylogeny (Supplementary Figure 3B). Together, our findings show that MSAD-1 and MSAD-2 sequences basically reflect the phylogenetic relationships between strains within the genus *Mycobacterium* except for some strains subject to LGT of their MSAD-1 gene, suggesting their pivotal role in the pathogenesis and evolution of *Mycobacterium* speciation.

### 3.5. MSAD-1 distribution between strains of *Mycobacterium avium* subspecies

**FIGURE 7:**
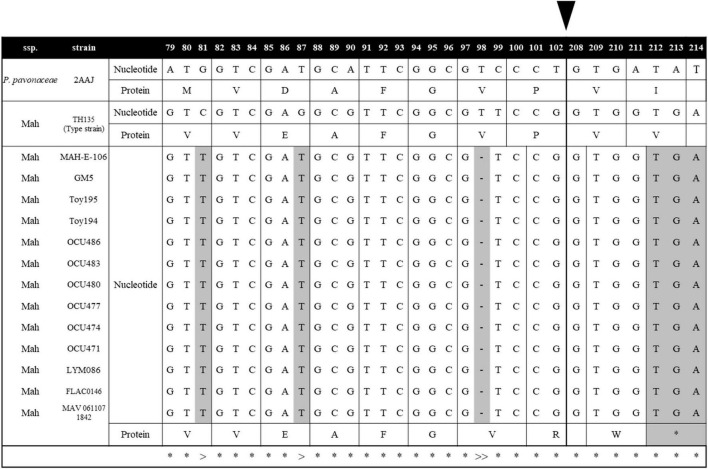
MSAD-1 sequence alignments of 13 *Mycobacterium avium* strains with truncated MSADs. The 13 *M. avium* strains with truncated MSADs have mutations at C81T and G87T and a one-letter gene deletion at 98T. The MSAD protein of *M. avium* consists of 130 amino acids, but the 13 strains above were truncated to 71 amino acids due to frameshift by deletion. The symbol “*” indicates that all mycobacterium nucleotides are the same. The symbol “>” represents the site where synonymous mutation occurred and the symbol “>>” where deletion occurs and a frameshift occurs. The black arrow indicates a skip from the 102nd nucleotide to the 208th nucleotide. This number corresponds to the codon number of the *P. pavonaceae* MSAD protein.

*Mycobacterium avium* (Ma) is one of the most virulent NTM species, causing a broad spectrum of diseases in humans and ruminant animals as a member of the MAC, and it consists of four subspecies, namely, *M. avium* (Maa), *M. hominissuis* (Mah), *M. paratuberculosis* (Map), and *M. silvaticum* (Mas) (Thorel et al., 1990). Due to their distinct pathogenic potentials, their subspecies separation has recently gained great attention (Turenne et al., 2007). In this study, to investigate MSAD distributions between strains of Ma subspecies, a total of 255 genome sequences of four Ma subspecies were analyzed using tBLASTn. Of note, none of the 49 Map strains and 1 Mas strain had the MSAD gene in their genomes (Table 1). Only some Maa strains and Mah strains have MSAD-1 orthologs in their genomes. Of the 43 Maa strains, only 17 strains (39.53%) have MSAD-1 genes, showing 99.0–100% sequence similarity values between strains. In particular, a type strain of Maa, ATCC 25291^T^, does not have MSAD in its genome. In the case of Mah, 104 of 162 strains (64.19%) have MSADs in their genomes, showing 98.7–100% sequence similarity values between strains (Table 1). Of note, among the 121 Ma strains with MSADs in their genomes, 13 (11 from Mah and 2 from Maa) have identical types of mutations in their MSAD genes, including a total of three mutations, two types of silent mutations, C81T and G87T, and a one-letter deletion at site 98T, which causes premature termination of the MSAD protein (Figure 7). Interestingly, 9 of 13 Ma strains with truncated MSADs were isolated from domestic pigs in Japan (Table 2; To et al., 2020; Komatsu et al., 2021), highlighting their potential roles in the pathogenesis or epidemiology of swine mycobacteriosis.

**TABLE 1:** Distribution of MSAD-1 among strains of *Mycobacterium avium* subspecies.

Subspecies name	MSAD protein sequence similarity (%)	Number of strains	
		MSAD	Non-MSAD
*Mycobacterium avium*	98.7–100	121	134
*M. avium* subsp. *avium*	99–100	17	26
*M. avium* subsp. *hominissuis*	98.7–100	104	58
*M. avium* subsp. *paratuberculosis*		0	49
*M. avium* subsp. *silvaticum*		0	1

None of the *M. avium* subspecies *paratuberculosis* and *silvaticum* strains have MSAD-1. Even among *M. avium* subspecies *avium* and *hominissuis*, the presence or absence of MSAD varies based on the strain of the species.

**TABLE 2:** Summary information of 13 *M. avium* strains with truncated MSAD-1 genes.

Subspecies	Strain	Accession_NCBI	Genome length (bp)	Accession_assembly	Country	Patient	CDS	Reference
*hominissuis*	MAH-E-106	NZ_LMVZ00000000	5111701	GCF_001610545.1	Germany	Soil	4,876	Unpublished
*hominissuis*	GM5	NZ_WEGX00000000	5037010	GCF_020055295.1	Japan	Swine	4,844	Komatsu et al., 2021
*hominissuis*	Toy194	NZ_WEGP00000000	5347524	GCF_020055155.1	Japan	Swine	5,001	
*hominissuis*	Toy195	NZ_WEGO00000000	5346468	GCF_020055125.1	Japan	Swine	5,013	
*hominissuis*	OCU480	NZ_WEHE00000000	5088946	GCF_020055405.1	Japan	Swine	4,792	
*hominissuis*	OCU474	NZ_WEHK00000000	5087878	GCF_020054555.1	Japan	Swine	4,792	
*hominissuis*	OCU477	NZ_WEHH00000000	5087664	GCF_020055495.1	Japan	Swine	4,749	
*hominissuis*	OCU486	NZ_WEGY00000000	5023805	GCF_020055345.1	Japan	Swine	4,703	
*hominissuis*	OCU471	NZ_WEHN00000000	4990913	GCF_020055565.1	Japan	Swine	4,683	
*hominissuis*	OCU483	NZ_WEHB00000000	4943024	GCF_020055385.1	Japan	Swine	4,624	
*hominissuis*	LYM086	NZ_FKJM00000000	5304361	GCF_900079545.1	Unknown	Unknown	5,105	Unpublished
*avium*	FLAC0146	NZ_NSFH00000000	5179834	GCF_002292595.1	United States	Homo sapiens	4,875	Unpublished
*avium*	MAV_061107_1842	NZ_JAOQ00000000	5320946	GCF_000523655.1	United States	Homo sapiens	4,981	Unpublished

Of these, nine strains were isolated from domestic pigs in Japan.

Together with our finding that there are no MSAD orthologs in the genome of Map strains, the finding that 13 of the 121 Ma strains with an MSAD-1 gene in their genome have mutated MSAD genes with premature translation termination further highlights the role of MSAD pseudogenization in species or subspecies differentiation of Ma.

### 3.6. Induction of pro-inflammatory cytokines in murine macrophage, J774A.1 cell by MSAD-1 protein of *Mycobacterium abscessus*

**FIGURE 8:**
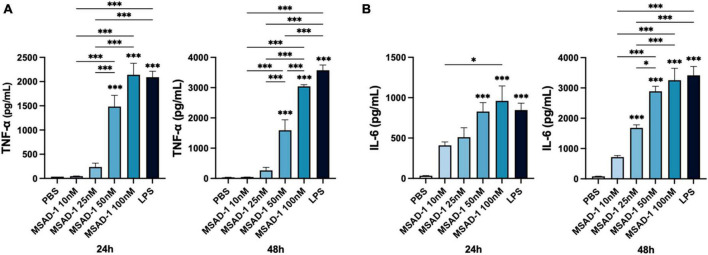
Induction of pro-inflammatory cytokines, TNF-α, and IL-6, in murine macrophage, J774A.1 cells, by MSAD-1 protein of *Mycobacterium abscessus*. **(A)** The levels of TNF-α induced by MSAD-1 were measured by ELISA in the culture medium after 24 or 48 h. **(B)** The levels of IL-6 induced by MSAD-1 were measured by ELISA in the culture medium after 24 or 48 h. Data represent means ± SEM (standard error of mean) from quadruplicate samples and are representative of at least two independent experiments. Statistical analysis was performed using one-way ANOVA with Tukey’s multiple comparisons test, and statistical significance are denoted by asterisks (*P < 0.05, ***P < 0.001). An asterisk above the bar indicates statistical significance compared to the PBS control group, and an asterisk with a line indicates statistical significance compared to the indicated group.

Our findings showing that loss of MSAD gene in the genome of pathogenic mycobacteria during their evolutionary adaptation prompt us to hypothesize that MSAD loss could contribute to their pathogenesis via evading immune responses of innate cells, macrophage, or dendritic cells. To address this hypothesis, we evaluated effect of MSAD-1 of *M. abscessus* on production of pro-inflammatory cytokines in murine macrophage, J774A.1 cells (Figure 8). We found that MSAD-1 treatment exerted enhanced productions of two inflammatory cytokines, TNF-α and IL-6 in J774A.1 cells in a dose dependent manner, suggesting that it could elicit immune response of innate cells in infection of mycobacteria.

## 4. Discussion

Comparative genomic analysis has revealed reductive genome evolution over the course of mycobacterial speciation, particularly in pathogenic slow-growing strains with strict host-associated lifestyles, including *M. tuberculosis* (Gagneux and Small, 2007; ten Bokum et al., 2008; Gagneux, 2018), *M. leprae* (Gómez-Valero et al., 2007; van Hooij and Geluk, 2021), and *M. ulcerans* (Stinear et al., 2007; Röltgen et al., 2012). Despite its extensive distribution throughout the biosphere, MSAD is not a housekeeping gene; thus, its distribution between mycobacterial strains with broad-spectrum lifestyles from environmental saprophytes to strict host-restricted pathogens could provide a deep understanding of their evolution and pathogenesis.

There are several noteworthy findings in this study. First, we found that there are no MSAD orthologs in the genome of more than half of the analyzed reference mycobacterial strains (105 of 192 strains, 54.7%) (Figure 3). In particular, highly virulent strains such as *M. tuberculosis*, *M. leprae*, *M. ulcerans, M. marinum*, Map, and *Mycobacterium kansasii*, which are regarded as overt human pathogens, do not have MSADs in their genomes (Supplementary Table 1), consistent with previous findings of their reductive genome evolution (Brosch et al., 2001; Veyrier et al., 2011; Wee et al., 2017). Moreover, we did not find any MSAD orthologs in any strains of Map, which is more virulent than other Ma subspecies, including Maa and Mah (Turenne et al., 2007) (Figure 7). In addition, we confirmed pseudogenization events in the MSAD genes of 13 strains of Maa and Mah by one-letter gene deletion causing frameshift events (Figure 7), further supporting our hypothesis of MSAD gene loss in more pathogenic mycobacteria.

Second, our SSN and phylogenetic analysis revealed two distinct MSAD types, MSAD-1 and MSAD-2, in *Mycobacterium* (Figures 2–5). We found that in 87 of 192 strains (45.3%), MSAD-1s (found in 57 sequences from 56 slow-growing strains and rapid-growing strains, two distinct MSAD-1s in *M. simiae*) and MSAD-2s (found in 43 strains in the *M. terrae* complex and rapid-growing strains) were distributed in an exclusive manner among *Mycobacterium* species. However, 12 rapid-growing strains, including three *M. chelonae* members, had both types of MSADs, MSAD-1 and MSAD-2, in their genomes (Supplementary Table 2). These findings suggest an evolutionary event from a common ancestor with both types of MSADs, possibly belonging to the rapid-growing group, into the strain with either a single MSAD-1 or a single MSAD-2 during *Mycobacterium* speciation. Both types of MSADs show distinct distributions among *Mycobacterium* strains. In particular, among slow-growing strains with an MSAD gene, the Tuberculosis-Simiae clade, including most pathogenic slow-growing strains, has only MSAD-1 (Figure 6), suggesting a pivotal role of MSAD-1 in the pathogenesis and evolution of pathogenic slow-grower. In contrast, the Triviale and Terrae clades have only the MSAD-2 type in their genomes (Figure 6), highlighting the role of MSAD-2 in their pathogenesis and evolution. In rapid-growers, the MSAD distribution is more complex than that in slow-grower. Rapid-growing strains with the MSAD gene could be divided into three groups according to their MSAD distributions: a group with only the MSAD-1 type, including three subspecies of *M. abscessus*, namely, *M. abscessus*, *M. massiliense* and *M. bolleti*, a group with only the MSAD-2 type, including most of the Fortuitum-Vaccae clade, and a group with both types of MSADs, MSAD-1 and MSAD-2, including three members of the *M. chelonae* subspecies (Figure 6 and Supplementary Table 1), suggesting distinct roles of the two MSAD types in pathogenesis and evolution in terms of respective rapid-growing groups. Given the close phylogenetic relationships between *M. chelonae* and *M. abscessus* strains (Adékambi and Drancourt, 2004; Tortoli et al., 2017), it is tempting to speculate on MSAD-2 loss during speciation from the common ancestor of two species with both types of MSADs into *M. abscessus*, which may have contributed to its pathogenesis.

Third, our data showed that the MSAD-1s of the slow-growers and rapid-growers and MSAD-2s have distinct primary signature sequences that can play a crucial role in their function. The MSAD-1s of all slow-growers have Arg-73, Arg-75, and Asp-37, which play a critical role in substrate reactions, and Trp-114, Phe-116, Phe-123, and Leu-128, which are essential for the hydrophobic wall, as shown in *P. pavonaceae* strain 170’s MSAD (PpMSAD) (Figure 5A and Supplementary Data 1; Almrud et al., 2005), suggesting a similar role of the MSADs of slow-growers with that of the latter. On the other hand, the MSAD-1s of rapid-growers have a primary signature sequence of Thr-72, Gln-73, and Tyr-123 (Guo et al., 2013) instead of a pair of arginines reacting with the substrate, distinct from the pattern in slow-growers, as shown in the MSADs of *C. bacterium* strain FG41 (FG41 MSAD) (Figure 5B and Supplementary Data 1; Guo et al., 2013), suggesting distinct functional roles or distinct evolutionary selection pressures between the MSAD-1s of slow-growers and rapid-growers. Interestingly, all the strains with MSAD-2 have primary sequences distinct from MSAD-1, suggesting distinct functional roles of MSAD-2s from slow-growers and rapid-growers MSAD-1s (Figure 5C and Supplementary Data 1). Further research is needed to determine the exact role of MSAD-2s in enzyme function.

Our phylogenetic analysis showed that MSAD-2 is more resistant than MSAD-1 to LGT events (Figure 6). Comparing the results with phylogenetic trees based on MSAD-1, MSAD-2, and *hsp65* sequences, we could not find any LGT events in 43 strains with MSAD-2, but 5 of 53 strains had MSAD-1s (9.4%) (the second MSAD-1 type of *M. simiae, M. dioxanotrophicus, M. farcinogenes, M. senegalense*, and *M. agri*), which may have been laterally transferred from another bacterial group (Figure 6A and Supplementary Table 3). Of these, 4 strains (*M. dioxanotrophicus, M. farcinogenes, M. senegalense*, and *M. agri*) belong to the rapid-growers of the Fortuitum-Vaccae clade. Interestingly, we found that while one (accession number BBX43605.1) of two MSAD-1 genes of *M. simiae* belongs to the clade of Tuberculosis-Simiae by MSAD-1-based phylogenetic analysis, the other MSAD-1 of *M. simiae* (accession number BBX40959.1) did not (Figure 6A). Our protein similarity network analysis indicated that it has sequence similarity with the MSAD from the *Methylobacterium phylum* (Supplementary Table 3), suggesting acquisition by *M. simiae* of the second MSAD-1 gene via LGT.

Our *in vitro* experiment using MSAD-1 protein indicated that MSAD-1 could evoke inflammatory response from innate cells in mycobacteria infection, suggesting that MSAD-1 loss in pathogenic mycobacteria could contribute into their chronic infection or pathogenesis via evading immune response of innate cell. However, the role of two distinct MSADs, MSAD-1 and MSAD-2, in mycobacterial pathogenesis or evolution must be proved in the future via further *in vitro* and *in vivo* studies using MSAD gene knock out or reinforced mutant.

## 5. Conclusion

In conclusion, our data revealed two distinct types of MSADs, MSAD-1 and MSAD-2, among strains in the *Mycobacterium* genus, but more than half of the strains, including strains of pathogenic mycobacteria such as *M. tuberculosis*, *M. leprae, M. marinum, M. ulcerans* and Map, have no MSAD orthologs in their genomes. Furthermore, in 13 *Ma* strains, MSAD-1 pseudogenization was found, suggesting MSAD-1 loss during host adaptation of pathogenic mycobacteria. Loss of MSAD during speciation could contribute to their pathogenicity via escape from host innate immune cells.

There are several limitations to this study. Study regarding the role of MSAD in mycobacteria evolution and pathogenesis is mainly focused on the bioinformatics prediction. Biochemical and structural evidence based on actual enzyme activities of MSAD-1 and MSAD-2 have not been introduced. So, these limitations should be addressed in the future.

## Data availability statement

The datasets presented in this study can be found in online repositories. The names of the repository/repositories and accession number(s) can be found in the article/Supplementary material.

## Author contributions

B-JK: Conceptualization, Data curation, Funding acquisition, Project administration, Validation, Writing – original draft, Writing – review and editing. DL: Conceptualization, Data curation, Investigation, Methodology, Software, Validation, Visualization, Writing – original draft, Writing – review and editing. DK: Data curation, Methodology, Software, Validation, Visualization, Writing – review and editing. HS: Conceptualization, Formal analysis, Writing – review and editing. SC: Data curation, Methodology, Validation, Visualization, Writing – review and editing.

## Footnotes

1
http://www.sbg.bio.ic.ac.uk/phyre2
