# Supplementary material for: Phylogenetic distribution of malonate semialdehyde decarboxylase (MSAD) genes among strains within the genus Mycobacterium: evidence of MSAD gene loss in the evolution of pathogenic mycobacteria
Source: Front Microbiol. 2023 Oct 13;14:1275616. doi: 10.3389/fmicb.2023.1275616 (PMC10606566; doi:10.3389/fmicb.2023.1275616)
Supplement: Supplementary file 1 [file Table_1.docx]

**Supplementary Table 1.** Information of genome sequencing project, accession numbers and the distribution of MSAD among 192 *Mycobacterium* reference strains used in this study.

| Species name | Strain | Accseion_NCBI | WGS | MSAD-1 | MSAD-2 |
| --- | --- | --- | --- | --- | --- |
| *Mycobacterium abscessus subsp. abscessus* | ATCC 19977 |  | MLCG | YES | - |
| *Mycobacterium abscessus subsp. bolletii* | CCUG 50184 |  | LDMY | YES | - |
| *Mycobacterium abscessus subsp. massiliense* | CCUG 48898 |  | AKVF | YES | - |
| *Mycobacterium agri* | JCM 6377 |  | BLKS | YES | - |
| *Mycobacterium ahvazicum* | AFP003 |  | FXEG | YES | - |
| *Mycobacterium aichiense* | DSM 44147 |  | JACKTJ | - | - |
| *Mycobacterium algericus* | DSM 45454 |  | MVHC | - | YES |
| *Mycobacterium alsense* | DSM 45230 |  | MVHD | - | - |
| *Mycobacterium angelicum* | DSM 45057 |  | MVHE | - | - |
| *Mycobacterium anyangense* | JCM 30275 | AP022620.1 |  | - | - |
| *Mycobacterium aquaticum* | RW6 |  | MVHF | - | YES |
| *Mycobacterium arabiense* | DSM 45768 |  | JACKVD | - | - |
| *Mycobacterium aromaticivorans* | JCM 16368 |  | BBHC | - | - |
| *Mycobacterium arosiense* | DSM 45069 |  | MVHG | - | - |
| *Mycobacterium arupense* | DSM 44942 |  | MVHH | - | YES |
| *Mycobacterium asiaticum* | DSM 44297 |  | MVHI | YES | - |
| *Mycobacterium aubagnense* | DSM 45150 |  | POTN | YES | YES |
| *Mycobacterium aurum* | DSM 43999 |  | JACKRV | - | - |
| *Mycobacterium austroafricanum* | DSM 44191 |  | CCAW | - | - |
| *Mycobacterium avium subsp. hominissuis* | MAHP-04-13 |  | LNBA | YES | - |
| *Mycobacterium avium subsp. paratuberculosis* | ATCC 19698 |  | AGAR | - | - |
| *Mycobacterium avium subsp. silvaticum* | ATCC 49884 |  | AYOC | - | - |
| *Mycobacterium bacteremicum* | DSM 45578 |  | MVHJ | - | - |
| *Mycobacterium boenickei* | JCM 15653 | AP022579.1 |  | - | YES |
| *Mycobacterium bohemicum* | DSM 44277 |  | LQOK | YES | - |
| *Mycobacterium botniense* | JCM 17322 |  | BLKW | - | - |
| *Mycobacterium bouchedurhonense* | DSM 45439 |  | JACKTG | YES | - |
| *Mycobacterium bourgelatii* | DSM 45746 |  | JACKSX | - | - |
| *Mycobacterium branderi* | DSM 44624 |  | MVHM | - | - |
| *Mycobacterium brisbanense* | JCM15654 |  | BCSX | - | YES |
| *Mycobacterium brumae* | DSM 44177 |  | JACKTW | - | - |
| *Mycobacterium canariasense* | CCUG 47953 |  | LQOL | YES | YES |
| *Mycobacterium celatum* | DSM 44243 |  | LQOM | - | - |
| *Mycobacterium celeriflavum* | DSM 46765 |  | JACKUE | - | - |
| *Mycobacterium chelonae* | ATCC 35752 |  | MLCH | YES | YES |
| *Mycobacterium chelonae subsp. bovis* | QIA-37 | CP010071.1 |  | YES | YES |
| *Mycobacterium chelonae subsp. gwanakae* | MOTT36W | CP031516.1 |  | YES | YES |
| *Mycobacterium chitae* | DSM 44633 |  | JACKSM | - | - |
| *Mycobacterium chlorophenolicum* | JCM 7439 |  | BCQY | - | - |
| *Mycobacterium chubuense* | NCTC10819 |  | UATB | - | YES |
| *Mycobacterium colombiense* | CECT 3035 |  | AFVW | YES | - |
| *Mycobacterium conceptionense* | CCUG 50187 |  | LQOP | - | YES |
| *Mycobacterium confluentis* | DSM 44017 |  | JACKUS | - | - |
| *Mycobacterium conspicuum* | DSM 44017 |  | LQOR | YES | - |
| *Mycobacterium cookii* | DSM 43922 |  | JACKUP | YES | - |
| *Mycobacterium cosmeticum* | DSM 44829 |  | CCBB | YES | - |
| *Mycobacterium crocinum* | DSM 45433 |  | JACKTV | - | - |
| *Mycobacterium diernhoferi* | DSM 43524 |  | JACKTT | - | - |
| *Mycobacterium dioxanotrophicus* | PH-06 | CP020809.1 |  | YES | YES |
| *Mycobacterium doricum* | CCUG 46352 |  | JACKUD | - | - |
| *Mycobacterium duvalii* | DSM 44244 |  | JACKUY | - | - |
| *Mycobacterium elephantis* | DSM 44368 |  | ATDN | - | - |
| *Mycobacterium engbaekii* | ATCC 27353 |  | LQOT | - | - |
| *Mycobacterium europaeum* | CSUR P1344 |  | CTEC | YES | - |
| *Mycobacterium fallax* | DSM 44179 |  | LQOJ | - | - |
| *Mycobacterium farcinogenes* | DSM 43637 |  | CCAY | YES | YES |
| *Mycobacterium flavescens* | DSM 43991 |  | JACKUL | - | - |
| *Mycobacterium florentinum* | DSM 44852 |  | LQOV | YES | - |
| *Mycobacterium fluoranthenivorans* | DSM 44556 |  | JAANOW | - | YES |
| *Mycobacterium fortuitum subsp. acetamidolyticum* | JCM6368 |  | BCSZ | - | - |
| *Mycobacterium fortuitum subsp. fortuitum* | DSM 46621 |  | ALQB | - | YES |
| *Mycobacterium fragae* | DSM 45731 |  | LQOW | - | - |
| *Mycobacterium franklinii* | DSM 45524 |  | MVHQ | YES | - |
| *Mycobacterium frederiksbergense* | DSM 45364 |  | JACKTH | - | - |
| *Mycobacterium gadium* | DSM 44077 |  | JACKRT | - | - |
| *Mycobacterium gastri* | DSM 43505 |  | LQOX | - | - |
| *Mycobacterium gilvum* | NCTC10742 |  | UGQM | - | - |
| *Mycobacterium goodii* | CCUG 58730 |  | JACKUB | YES | - |
| *Mycobacterium gordonae* | DSM 44160 |  | LQOY | YES | - |
| *Mycobacterium grossiae* | GK |  | MCHX | - | - |
| *Mycobacterium hackensackense* | DSM 44833 |  | JACKUC | - | - |
| *Mycobacterium haemophilum* | DSM 44634 |  | JACKVB | - | - |
| *Mycobacterium hassiacum* | DSM 44199 |  | ARBU | - | - |
| *Mycobacterium heckeshornense* | DSM 44428 |  | JACKTA | - | - |
| *Mycobacterium heidelbergense* | DSM 44471 |  | MVHR | - | - |
| *Mycobacterium helveticum* | 16-83 |  | VMQU | - | - |
| *Mycobacterium heraklionense* | JCM 30995 | CP080997.1 |  | - | YES |
| *Mycobacterium hiberniae* | DSM 44241 |  | JACKSF | - | YES |
| *Mycobacterium hippocampi* | DSM 45391 |  | JACKSE | - | - |
| *Mycobacterium holsaticum* | DSM 44478 | CP080998.1 |  | - | - |
| *Mycobacterium houstonense* | ATCC 49403 |  | FJVO | - | - |
| *Mycobacterium icosiumassiliensis* | 8WA6 |  | FJVP | - | YES |
| *Mycobacterium immunogenum* | DSM 45595 |  | JACKUV | YES | - |
| *Mycobacterium insubricum* | DSM 45132 |  | JACKRM | - | - |
| *Mycobacterium interjectum* | DSM 44064 |  | LQPB | YES | - |
| *Mycobacterium intermedium* | DSM 44049 |  | MVHT | - | - |
| *Mycobacterium intracellulare subsp. chimaera* | DSM 44623 |  | LQOO | YES | - |
| *Mycobacterium intracellulare subsp. intracellulare* | ATCC 13950 |  | ABIN | YES | - |
| *Mycobacterium intracellulare subsp. yongonense* | 05-1390 | CP003347.1 |  | YES | - |
| *Mycobacterium iranicum* | DSM 45541 |  | LQPC | - | - |
| *Mycobacterium kansasii* | ATCC 12478 | CP006835.1 |  | - | - |
| *Mycobacterium komossense* | DSM 44078 |  | JACKTY | - | - |
| *Mycobacterium koreense* | KCTC 19819 |  | NCXO | - | YES |
| *Mycobacterium kubicae* | JCM 13573 |  | BLKU | YES | - |
| *Mycobacterium kumamotonense* | DSM 45093 |  | MVHU | - | YES |
| *Mycobacterium kyogaense* | NCTC 1659 |  | QJUA | - | YES |
| *Mycobacterium kyorinense* | DSM 45166 |  | LQPE | - | - |
| *Mycobacterium lacus* | DSM 44577 |  | LQPF | - | - |
| *Mycobacterium lehmannii* | CECT 8763 |  | NKCN | - | - |
| *Mycobacterium lentiflavum* | ATCC 51985 | CP092423.1 |  | - | - |
| *Mycobacterium leprae* | TN | AL450380.1 |  | - | - |
| *Mycobacterium litorale* | DSM 45785 |  | JACKVO | - | - |
| *Mycobacterium llatzerense* | MG13 |  | LXOV | - | YES |
| *Mycobacterium longobardum* | DSM 45394 |  | LQPG | - | - |
| *Mycobacterium lutetiense* | DSM 46713 |  | JAGIOP | - | YES |
| *Mycobacterium mageritense* | DSM 44476 |  | CCBF | YES | - |
| *Mycobacterium malmoense* | ATCC 29571 | CP080999.1 |  | - | - |
| *Mycobacterium manitobense* | DSM 44615 |  | JACKSJ | - | - |
| *Mycobacterium mantenii* | DSM 45255 |  | MVHW | YES | - |
| *Mycobacterium marinum* | NCTC2275 |  | PEDD | - | - |
| *Mycobacterium marseillense* | DSM 45437 |  | JACKVF | - | - |
| *Mycobacterium minnesotense* | DSM 45633 |  | JACKRY | - | YES |
| *Mycobacterium monacense* | DSM 44395 |  | MVIA | - | - |
| *Mycobacterium montefiorense* | DSM 44602 |  | JACKVL | - | - |
| *Mycobacterium moriokaense* | DSM 44221 |  | QQBJ | - | - |
| *Mycobacterium mucogenicum* | CSUR P2099 |  | CYSI | - | YES |
| *Mycobacterium murale* | JCM 13392 |  | BLKT | - | - |
| *Mycobacterium nebraskense* | DSM 44803 |  | LQPH | YES | - |
| *Mycobacterium neoaurum* | DSM 44074 |  | CCDR | - | - |
| *Mycobacterium neumannii* | CECT 8766 |  | NKCO | - | - |
| *Mycobacterium neworleansense* | ATCC 49404 |  | CWKH | - | YES |
| *Mycobacterium nivoides* | DL90 | CP034072.1 |  | - | YES |
| *Mycobacterium nonchromogenicum* | DSM 44164 |  | LQPI | - | YES |
| *Mycobacterium noviomagense* | DSM 45145 |  | JACKRQ | - | - |
| *Mycobacterium novocastrense* | JCM18114 |  | BCTA | - | - |
| *Mycobacterium obuense* | DSM 44075 |  | JYNU | - | - |
| *Mycobacterium ostraviense* | 241/15 |  | NKRE | - | - |
| *Mycobacterium palauense* | CECT 8779 |  | NVQF | - | - |
| *Mycobacterium pallens* | JCM 16370 |  | BBHE | - | - |
| *Mycobacterium palustre* | DSM 44572 |  | LQPJ | - | - |
| *Mycobacterium paraense* | IEC26 |  | LQPM | YES | - |
| *Mycobacterium paraffinicum* | DSM 44181 |  | JACKUR | YES | - |
| *Mycobacterium parafortuitum* | CCUG 20999 |  | MVID | - | - |
| *Mycobacterium paragordonae* | JCM 18565 |  | BLKX | YES | - |
| *Mycobacterium parakoreense* | DSM 45575 |  | JACKUQ | - | YES |
| *Mycobacterium parascrofulaceum* | ATCC BAA-614 |  | ADNV | - | - |
| *Mycobacterium paraseoulense* | DSM 45000 |  | MVIE | YES | - |
| *Mycobacterium parmense* | DSM 44553 |  | LQPO | - | - |
| *Mycobacterium peregrinum* | DSM 43271 |  | LQPP | - | YES |
| *Mycobacterium persicum* | AFPC-000227 |  | MVIF | - | - |
| *Mycobacterium phlei* | DSM 43239 |  | ANBP | YES | - |
| *Mycobacterium phocaicum* | DSM 45104 |  | POTM | YES | YES |
| *Mycobacterium porcinum* | DSM 44242 |  | JACKVC | - | YES |
| *Mycobacterium poriferae* | DSM 44585 |  | JACKUH | - | - |
| *Mycobacterium pseudoshottsii* | JCM 15466 |  | BCND | - | - |
| *Mycobacterium psychrotolerans* | JCM 13323 | AP022574.1 |  | - | - |
| *Mycobacterium pulveris* | DSM 44697 |  | JACKSQ | - | - |
| *Mycobacterium pyrenivorans* | DSM 44605 |  | JACKSH | - | - |
| *Mycobacterium rhodesiae* | DSM 44223 |  | MVIH | - | - |
| *Mycobacterium riyadhense* | DSM 45176 |  | LQPQ | - | - |
| *Mycobacterium rufum* | JS14 |  | JROA | - | - |
| *Mycobacterium rutilum* | JCM 16371 |  | BBHF | - | - |
| *Mycobacterium salmoniphilum* | SC |  | MAFR | YES | YES |
| *Mycobacterium saopaulense* | CCUG 66554 |  | MVII | YES | - |
| *Mycobacterium saskatchewanense* | DSM 44616 |  | LQPR | YES | - |
| *Mycobacterium scrofulaceum* | DSM 43992 |  | MVIJ | YES | - |
| *Mycobacterium sediminis* | DSM 45643 |  | JACKUW | - | - |
| *Mycobacterium senegalense* | DSM 43656 |  | JACKUT | YES | YES |
| *Mycobacterium senriense* | TY59 | AP024828.1 |  | YES | - |
| *Mycobacterium senuense* | JCM 16017 |  | BLKV | - | - |
| *Mycobacterium seoulense* | DSM 44998 |  | JACKVP | YES | - |
| *Mycobacterium septicum* | DSM 44393 |  | CBMO | - | YES |
| *Mycobacterium setense* | DSM 45070 |  | JTJW | - | - |
| *Mycobacterium sherrisii* | ATCC BAA-832 |  | LQPT | YES | - |
| *Mycobacterium shigaense* | UN-152 |  | MWON | YES | - |
| *Mycobacterium shimoidei* | DSM 44152 |  | LQPU | - | - |
| *Mycobacterium shinjukuense* | CCUG 53584 |  | MVIK | - | - |
| *Mycobacterium simiae* | JCM 12377 | AP022568.1 |  | YES | - |
| *Mycobacterium smegmatis* | VKM Ac-1171 |  | JAMZOD | - | - |
| *Mycobacterium sphagni* | ATCC 33027 |  | NOZR | - | - |
| *Mycobacterium stephanolepidis* | NJB0901 | AP018165.1 |  | YES | YES |
| *Mycobacterium stomatepiae* | DSM 45059 |  | JACKSO | YES | - |
| *Mycobacterium syngnathidarum* | 27335 |  | MLCL | - | YES |
| *Mycobacterium szulgai* | DSM 44166 |  | LQPW | - | - |
| *Mycobacterium talmoniae* | ATCC BAA-2683 |  | PPEA | YES | YES |
| *Mycobacterium terrae* | CIP 104321 |  | LQPX | - | YES |
| *Mycobacterium terramassiliense* | AB308 |  | FTRV | YES | - |
| *Mycobacterium thermoresistibile* | DSM 44167 |  | JACKTR | - | - |
| *Mycobacterium timonense* | CCUG 56329 |  | JACKSY | YES | - |
| *Mycobacterium tokaiense* | NCTC10821 |  | UGQT | - | - |
| *Mycobacterium triplex* | DSM 44626 |  | CCAU | - | - |
| *Mycobacterium trivialis* | DSM 44153 |  | LQPZ | - | YES |
| *Mycobacterium tuberculosis* | H37Rv |  | JLDD | - | - |
| *Mycobacterium tuberculosis variant microti* | Maus IV | LR882497.1 |  | - | - |
| *Mycobacterium tusciae* | DSM 44338 |  | MVIM | - | - |
| *Mycobacterium ulcerans* | ATCC 33728 | AP017635.1 |  | - | - |
| *Mycobacterium vaccae* | NBRC 14118 |  | BCRS | - | - |
| *Mycobacterium vanbaalenii* | DSM 7251 |  | JACKSD | - | - |
| *Mycobacterium virginiense* | DSM 100883 | CP092430.1 |  | - | YES |
| *Mycobacterium vulneris* | DSM 45247 |  | CCBG | - | YES |
| *Mycobacterium wolinskyi* | ATCC 700010 |  | LQQA | YES | - |
| *Mycobacterium xenopi* | NCTC10042 |  | UATA | - | - |

**Supplementary Table 2.** The 100 mycobacterial MSAD DNA sequences used in this study and their GenBank accession Number.

| Species Name | Accession Number | DNA region | Protein ID | DNA sequence |
| --- | --- | --- | --- | --- |
| *Mycobacterium abscessus* subsp. *abscessus* ATCC 19977 | MLCG01000005.1 | 297634-298041 | BBX92590.1 | TGCGCCTGCGGCGGGCACTGCGAGCTCACCGGTCACGTACTGCGCGTGTCCGAAGCCGAAAGACCAATCCTGAGGGCCATTTTCGCTGATACCGACGAACAGGTCCGCACCGGTCACACCGACAGCACCGAGCCGGTCTGCCAGCTCTGCGAAGATCCGTTGCTTGGTCTCGGTTGTGCGGCCGGTCTGGGTGAAGATATGCACGATCACCACGCTGCGCGACCGCCGGAATCCCAAACCGGCGTCCTCGGCGATGATGTCGCCCGCGTCGTGTGCGGTGATGATCTGAAAACGATCTCGGGCAGGGATCTTCAAGACTGCGACGAGGCCCTCGTGCACGCCGTCGGCGATCGCGCGTTGTTCTGCCGGGGACCGATCCGCGGTGAGGTCGATGCGCACCAATGGCAT |
| *Mycobacterium abscessus* subsp. *bolletii* CCUG 50184 | LDMY01000013.1 | 115141-115548 | TPF66183.1 | ATGCCATTGGTGCGCATCGACCTCACCGCAGATCGGTCCCGTGCAGAACAACGCGCGATCGCCGACGCCGTGCACGACGGCCTCGTCGCAGTCTTGAAAATCCCTGCCCGAGATCGCTTTCAGATCATCACCGCGCACGGTGCGGGTGACATCATCGCCGAGGACGCCGGCTTGGGATTCCGGCGGTCGCGCAGCGTGGTGATCGTGCACATCTTCACCCAGACCGGGCGGACAACCGAGACCAAGCAACGAATCTTCGCAGAGCTGGCAGACCGGCTCGGTGCTGTCGGCGTGAACGGGGCGGACCTGTTCGTCGGTATCAGCGAAAATGGCCCTCAGGACTGGTCTTTCGGCTTCGGACACGCGCAATATGTGACCGGTGAGCTCGCGGTGCCCGCCGCAGGCGCA |
| *Mycobacterium abscessus* subsp. *massiliense* CCUG 48898 | AKVF01000004.1 | 65152-65559 | EIV66470.1 | TGCGCCTGCGGCGGGCACCGCGAGCTCACCGGTCACATATTGCGCGTGTCCGAAGCCGAAAGACCAGTCCTGAGGGCCATTTTCGCTGATACCGACGAACAGGTCCGCCCCGTTCACGCCGACAGCACCGAGCCGGTCTGCCAGCTCTGCGAAGATTCGTTGCTTGGTCTCGGTTGTCCGCCCGGTCTGGGTGAAGATGTGCACGATCACCACGCTGCGCGACCGCTGGAATCCCAAGCCGGCGTCCTCGGCGATGATGTCACCCGCACCGTGCGCGGTGATGATCTGAAAGCGATCTCGGGCAGGGATTTTCAAGACTGCGACGAGGCCGTCGTGCACGGCGTCGGCGATCGCGCGTTGTTCTGCACGGGACCGATCTGCGGTGAGGTCGATGCGCACCAATGGCAT |
| *Mycobacterium agri* JCM 6377 | BLKS01000001.1 | 2321-2722 | GFG48563.1 | GGCGCGAACCTACAGTCGACCTTCGACGAACTGCGCGATGCCGTTACCGAAGGACCAATCCTCACGCGCGGTTTCGACAATGGAGACGATCAAATTCGATGGTTTCAAGCCGGTTTGATCGCGAAGACGGTCAGCAAGTGCCCGGTACGCCGCTTGCTTTTGGTCGCGTGTTCGACCCTGCTGAAAGATCTGCAAGATGACGACGTCATCAGTCCGCTCGAACCCGAGCCCGGTGTCCTCGGCGATCATCTGGCCCGGCTTGTGCTCGGTGATGATTTGATAGCGGTCCCCATCGGGTGCAGCGAAGCACTCGCTCATTACCTGCTGGACGGTATCCGCCAGTCGGGTGAGTTGTTCTGGCGTACGCCGGGCTTCGGTGACGTGAATGTGGACCAGTGGCAT |
| *Mycobacterium ahvazicum* AFP003 | FXEG02000003.1 | 381064-381450 | SOX54237.1 | GAGTTCCCCGGTGAGGAACTGCGCCCGACCGTGGCCGAAAGACCAGTCCTCGTCGCTATTTTCGGTGATCGATACGATCAGATCGGCCGGATCGACGCCACACCGCTCGGCGAGGTTGGCCGCCAGCAACTCGTAGAACCGTTCCTTCATGGCGCGGGTGCGCTGGCGACTGACCACGTGCACGATCACCAGCCGCGCCGAGCGGTCGATGCCCAGACCGGTGTCCCAGGCCACGATCTCGTGCGCGGGATGGGTGCGCACCACCTGGTAGCGGTCACCGGGCGGGACCGCAAACGCTCCGATGACGGCATCATGCGCGGCGTCCAGCAACGCCTGGACCTCCGAGGGGGTGCGCCCTTCGATCAGGTCGATGTACAACAACGGCAT |
| *Mycobacterium algericus* DSM 45454 | MVHC01000002.1 | 281139-281531 | OQZ99192.1 | CACCCGAACGTTGAACCCCAACTCGACGTCGGATGCGGCCTGCCCGCCGCGGATCCCCCAGTTCTCCCGGTCCACCTCGTGCAGCGTGATCATCACGTGGTCGATCGGGATACCCAGCGTCGCAAGGCGTTCGACGATCTCCGCGTAGAGTTTCCGCTTGGCTTCCAGGGTGCGGCCGGTGAAGCAGTCGATCGAGACCAGAGTGCGGTACTCGGGTCGGGTGAGTTTTTCCGGGACTGCGAACCGGTGCGGCTCATGAACCACCAGCCGGACGTTCTTGTCGTCGGCGGGCGTCTGGAAAGCCGCCACCAGCGCGCCGTGAACAGCGTCGATGATGGCGACTTCCTCGGCTTGCGAGTAGCGGCGGCGAACCTCGATCAGCACGGTCGGCAT |
| *Mycobacterium aquaticum* RW6 | MVHF01000068.1 | 647-1039 | ORA22535.1 | GTGCCGAGCTCACTGATCGAGGTCCGCAAGGGCTACACCGCCACCGAAGAGGTGGCTCTCATCGATGCCGTGCATGCCGCGCTCGTCGCTGCCTTCCAGATTCCCGAGGAAGACAAGCACGTGCGGCTGGTTTGCCACGAGCCGCATCGGTTCGCGTACTCACCGAAGCTCGCGAACCCGGATCTGTACACGCTCGTCACCATCGATTGCTTCGTGGGCCGCTCGGTGCAGGCCAAGCGCAATCTCTACGGCGAGATCGTCAGTCGCTTGGGCGCCTTCGGAATTCCGGCCGACCACATCACGATCCTGCTGCGTGAGAGTGCGCTGGAGAACTGGGGTGTGCGGGGCGGTCAGGCGGCCTGTGACGTTGATCTGGGATTCGACATCAACGTC |
| *Mycobacterium arupensis* DSM 44942 | MVHH01000002.1 | 95783-96175 | ORA00875.1 | CACCTGAACGGTGAAGCCCAGATCGACGTCCGACGCCGCCTGGCCGCCCCGGATGCCCCAATTGTCGCGGTCAGACTCGTGCAGGGTGATCATCACGTGGTCACGTGGAATACCAAGTTCGGCAAGGTTTTCGACGATTCCGGCGTAGAGCAGTCGCTTGGCCTCCACCGACCGCCCAGAGAAACAGTCGATGGAGACCAGGGTCCGATACTCGGGCTGGGCGAGCTGAGCGGGTACCACGAAGCGGTGCGGCTCGTGCACCACCAGCCGCACGTTCTTGTCCTTGGCCGGTATCTGGAACGCGGTCACCAGCGCGCCGTGAACGGCGTCGATGATCGCGACTTCCTCGGCCTGGGAGTAGTGGCGGCGAACCTCGATCAAGACAGTTGGCAT |
| *Mycobacterium asiaticum* DSM 44297 | MVHI01000063.1 | 12509-12874 | ORA08830.1 | CGCCCGACCGTGTCCGAAAGACCAGTCCTCGTCGTCGTTTTCGGTGATCGACACGATCAGGTCCGCCGGGTCCTGCCCGCATCGTTCGGCGAGTTTGGCCGCCACCCGCTCATAGAACTTCTGCTTGAGCTCGCGCGGCCGGCGTCGACTCACCACGTGCAGTATGACCAAGGCGTCGGTGCGATCAATGCCCAGACCGGTGTCCTGCGCGACGATCTCGTGGGCGGGATGGCTGCGCACCACCTGATAGCGATCGCGCTGTGGCACTCCGAACGCCTCGACCACCGCTTCGTGGATGGCGTCCAACAACACCCGAACCTCCACGGGCGTCCGCCCCTGGACAATGTCGATGTACAGCAGTGGCAT |
| *Mycobacterium aubagnense* DSM 45150 | POTN01000014.1 | 267234-267626 | TLH58201.1 | ATGCCGAGTTCACTGATCGAAGTACGCCGTCAGTATCGCCAAGCCGACGAGGCCGCCATCATCGACGCCGTCCATGATGCGCTGGTGGCCGCGTTCCACATCCCGGTCGGTGACAAGCACGTCCGGCTGCTGGTCCATGAACCGCACCGGTTTTCGCACGCACCGCAGCTGGCGCACCCCGAGCGGTACACGCTGGTGACCATCGACTGTTTCGCCGGCCGTTCGGTGGACGCCAAGCGCGCACTGTACCGCGAGATCGCGGGCCGCCTTGCCGCGTTCGGCATTCCGGCCGACCACGTCACGATCCTGCTGCGCGAGAGCGCGCTCGAGAACTGGGGTATCCGCGGCGGGCACGCGGCCTGCGACGTCAACCTCGGCTTCGACGTGAATGTC |
| *Mycobacterium aubagnense* DSM 45150 (2) | POTN01000041.1 | 6238-6627 | TLH66925.1 | ATGCCACTGCTGAACTTCACCGTCATCCGGGGCCGGACCTCCGAGCAGGTGCGGCAACTGCTCGACAGCGCACACCGCGCCGTCGTGGACGCCTTCGGAGTACCTGAGCGTGACCGCTACCAGATTGTCGATGTGCGCGAACCCGACGACGTGGTCGCGCTCGACACCGGGCTGGGATTCGAACGGTCCGACCGGCTGGTCATCATCGAGGTGGTCAGCCGGCGCCGCACTGCAGAACAGAAGCAACGGTTCTACGAACTGCTGGCCGAGAACCTACATCGCGACTGTGGATTGGACGCCACAGACCTCATCGTCGCAATCACCGAGAACGGCGATGCCGACTGGTCGTTCGGCGCCGGGCGCGCGCAATTCGTCACAGGTGAGCTGACC |
| *Mycobacterium avium* subsp. *hominissuis* MAHP-04-13 | LNBA01000124.1 | 25-414 | WP_011725047.1 | CGTGAGTTCGCCGGTCAGGAACTGAGCCCTCCCGTGGCCGAAAGACCAGTCCTCGTCGTCGTTTTCGGTGATCGAGACGATCAGGTCGGCGGGGTCGAGCCCGCACCGGCCGGCGAGGCGCGACGCCAGCAGCTCATAGAACTTCTGCTTGAGCTCACGCGGCCGCCGTCGGCTCACCACGTGCAAGACCACCTGGCGAGACGACCGATCGATGCCCAGACCGGTGTCCAGCGTAACGATTTCGTGTGCCGGGTGGGTATGCACCACCTGGTAGCGATCCCGCGGCGGAACGCCGAACGCCTCGACGACCGTGTCGTGGATCGCGTCCAGCAGGGCACTGACCTCCGAGGGCGTCCGACCCTCGATGAGGTCGATATACAACAGCGGCAT |
| *Mycobacterium boenickei* JCM 15653 | AP022579.1 | 4430752-4431144 | BBX92590.1 | ATGCCCAGCACGCTCATCGAGGTCAGGCGCCAGTACACGGAAGCCGAAGAGGTGGCGATCATCGACGCGGTGCACAACGCGCTGGTGGTTGCGTTCCAGATACCCTCCGGTGACAAGCACGTTCGGCTGGTATCCCATGTACCACATCGGTGCTCGCATTCACCCGGACTGGCTCACCCCGAGCTGTACACACTTGTCGCGATCGACTGCTTTGCCGGTCGATCGGTCCAGGCCAAGCGAAACCTCTACCGGGAGATCGTCACTCGCCTGGAGACACTGGGTATCCCGGCCGATCATGTCACGATCGTCCTGCGTGAGAGCGCGCTCGAGAACTGGGGCGTTCGTGGCGGGCGGGCCGCGTGCGATGTCGATCTGGGTTTCGACGTCAACGTC |
| *Mycobacterium bohemicum* DSM 44277 | LQOK01000028.1 | 226618-227007 | ORU99212.1 | ATGCCACTTATCTACGTCGACCTCATCGAGGGCCGAACGCAGTCGGAGGTCGCGCGGCTGCTGGACGCCATTCACGACGCCGTGGTCGAAGCGTTCGGGGTGCCGCCGCGCGACCGCTATCAGGTGGTGCGCACCCATCCCGCCGCCGAGATCGTCGCCCTCGACACCGGTCTGGGCATCGACCGCACGCCGCGGCTGGTCATCGTGCACGTGGTGAGCCGGCGGCGCGAACGCGCGATGAAGGAGCAGTTCTTCAAGGTGCTGGCGTCCAATCTGGACGTCCGGTGCGGCATCGACCCGGCCGACCTGATCGTCTCCATCACCGAAAACGGCGACGAGGACTGGTCATTCGGCCACGGCCGGGCGCAATTCCTCACCGGAGAGCTGACA |
| *Mycobacterium bouchedurhonense* DSM 45439 | JACKTG010000022.1 | 96257-96646 | MCV6989703.1 | ATGCCGCTGTTGTATATCGACCTCATCGAGGGTCGGACGCCCTCGGAGGTCAGTGCCCTGCTGGACGCGATCCACGACACGGTCGTCGAGGCGTTCGGCGTTCCGCCGCGGGATCGCTACCAGGTGGTGCATACCCACCCGGCACACGAAATCGTTACGCTGGACACCGGTCTGGGCATCGATCGGTCGTCTCGCCAGGTGGTCTTGCACGTGGTGAGCCGACGGCGGCCGCGTGAGCTCAAGCAGAAGTTCTATGAGCTGCTGGCGTCGCGCCTCGCCGGCCGGTGCGGGCTCGACCCCGCCGACCTGATCGTCTCGATCACCGAAAACGACGACGAGGACTGGTCTTTCGGCCACGGGAGGGCTCAGTTCCTGACCGGTGAACTCACG |
| *Mycobacterium brisbanense* JCM15654 | BCSX01000019.1 | 107418-107810 | GAS87603.1 | GTGCCGAGTTCGCTGATCGAGGTTCGCAAGCCCTACACGGTTGCCGAAGAGGTGGCCCTCATCGATGCCGTGCATGCCGCACTTGTCGCTGCTTTCCAGATTCCCGCGGAAGACAAGCACGTGCGGCTGGTGAGCCATGAGCCGCATCGGTTCGCGTATTCGCCGAAGCTCGCGAATCCCGATCTGTATACGCTCGTCACCATCGACTGCTTCGTGGGCCGCTCGGTGCAGGCCAAGCGCAATCTATACAGCCAGATCGTCAATCGCTTGGGGGACTTCGGAATTCCGGCCGACCACATCACCATCCTGCTGCGCGAGAGCGCGCTCGAGAACTGGGGCGTCCGAGGCGGACAGGCGGCCTGCGACGTTGATCTGGGCTTCGACGTCAACGTC |
| *Mycobacterium canariasense* CCUG 47953 | LQOL01000034.1 | 82932-83324 | ORU97003.1 | CACGTTGACATCGAAGCCCAGGTCGACGTCGCAGGCCGCTTGACCGCCGCGGATACCCCAGTTCTCCAGGGAGCTCTCCCGCAGCAGGATGGTGACGTGGTCGCGCGGAATGCCCAAGGCGTGCAGCGCGTCGGTGATCTCGCGGTAGAGGTTGCGCTTGGCCTGGACCGTGCGGCCGGCGAAACAATCGATGCCGACGAAGGTGTACAGCTCCGGTGTCGCGAGGCCGGGTGGTACAGCGAACCGGTGCGGTTCGTGAGACACCAGCCGAACGTGCTTGTCGACAACAGGGATCCGGAATGCCGCTACCAGCGCGCAGTGCACTGCGTCGATGATGGCGCCTTCTTCGGCTGGCGTGTATCGCCGTCGGACTTCGATGATGGTGCTGGGCAT |
| *Mycobacterium canariasense* CCUG 47953 (2) | LQOL01000099.1 | 4541-4852 | ORV10244.1 | GCCATCGCCGACGCCGTTCATCGTGCCGTCGTGGACGTGCTCGGCATTCCGGAGCGCGACCGCTTCCAGATTGTCACCACTCACCGGCCCGGCGAGATCGTGGCTCTGGACGCAGGTCTGGGCTTCGACCGCTCACCCGATGTGGTGATGATCCACATCTTCACGCAGGCGGGCCGCTCGGATACCACCAAACGGGACCTGTTCGCCCGGATCGCCGAGCGCCTGGCCGGGGTCGGCGTCGACGGGCGCGACGTCTTCGTCGGGATCACCGAGAACGGAGCGCAGGATTGGTCGTTCGGCTTCGGCAAGGCC |
| *Mycobacterium chelonae* subsp. *chelonae* ATCC 35752 | MLCH01000004.1 | 2244626-2245018 | OLT81431.1 | ATGCCCAGTTCAGTAGTCGAGGTCCGTCGGCGATACATCGAGGCCGAAGAGGTGGCGATCATCGATGCGGTCCACGGCGCGCTGGTCTCTGCCTTTCAGATACCGGAAGGGGACAAGCATGTACGCCTTGTCGTGCATGAACCCCACAGGTTTTCGCACGCGCCAAACCTGGCCCGGCCGGAGCTGTACACCTTTGTTTCCATCGACTGCTTCGCGGGCCGATCGATAGGGACGAAACGAAACCTCTATGCCGAGATTGTGAAACGGCTTTCCTTGCTGGGGATACCGCCGGATCACGTCACCATTGTGCTGCGGGAGAGTGCGACGGAGAATTGGGGAATCCGTGGAGGTCAGGCGGCCTGCGATATCGACCTGGGTTTCGATGTGAACGTC |
| *Mycobacterium chelonae* subsp. *chelonae* ATCC 35752 (2) | MLCH01000004.1 | 850762-851169 | OLT83119.1 | GCTCGCCGCGGCGGGCACAGCCAGCTCACCGGTAACGTACTGCGCGTTACCGAAGCCGAAAGACCAATCTTGCGGCCCATTTTCGCTGATCGCCACGAAAAGATCCGCGCCCGCCACATCGACGGCGGCCAGCCTGTTGGCCAGCTCGGCGAAGATCCGCTGCTTGGTCTCCGCGGTACGGCCGGCCTGGGTGAAGATGTGCACGATCACCACACGGGCCGATCGCGTGAACCCCAGTCCCGCATCCTCTGCGATGATGTCGGCGGCCTCATGCCCCGTGATGATCTGAAATCGGTCTCGTGCAGGGATTTTCAACACTTCGACCAAGGCGTCATGCACGGCGTCGGCGATGGCGCGTTGCTGATCACGTGACCGGTCGGAAGTAACGTCGATACGCACTAGTGGCAT |
| *Mycobacterium chelonae* subsp. *gwanakae* strain MOTT36W | CP031516.1 | 168823-169230 | AYM40275.1 | ATGCCACTAGTGCGTATCGACTTAACCTCTGACCGGCCACGCGATCAGCAACGCGCCATCGCCGACGCCGTGCATGACGCTCTGGTCGAAGTGTTGAAAATCCCTGTACGAGACCGATTTCAGATCATCACGGCGCATGAGGCCGCAGACATCATCGCAGAGGATGCGGGACTGGGCTTCGCGCGATCGTCCCGTGTGGTGATCGTGCACATCTTCACGCAGACCGGCCGTACCGTGGAGACCAAGCAGCAGATCTTCTCGGAGCTGGCCGACAGGCTGGCTGCCGTCGATGTGGCGGGCGCGGATCTTTTCGTGACGATCAGTGAAAATGGGCCGGAGGATTGGTCTTTCGGTTTCGGTAACGCGCAGTACGTTACCGGTGAGCTGGCTGTGCCCGCCGCAGCGAGC |
| *Mycobacterium chelonae* subsp. *gwanakae* strain MOTT36W (2) | CP031516.1 | 3840385-3840741 | AYM44870.1 | CGCCTGACCTCCACGGATTCCCCAATTCTCCGTCGCACTCTCCCGCAGCACAATGGTGACGTGATCCGGCGGTATCCCCAGTAAGGAAAGTCGTTTCACAATCTCGGCATAGAGGTTTCGTTTCGCCCCTATCGAGCGGCCCGCGAAGCAGTCGATGGAAACAAAGGTGTACAGCTCCGGCCGGGCCAGGTTTGGCGCGTGCGAAAACCTGTGGGGTTCATGCACGACAAGGCGTACATGCTTGTCCCCTTCCGGTATCTGAAAGGCAGCGACCAGCGCGCCGTGGACCGCATCGATGATCGCTACCTCTTCGGCCTCGGCGTATCGCTGACGGACCTCGACCACTGAACTGGGCAT |
| *Mycobacterium chelonae* subsp. *bovis* QIA-37 | CP010071.1 | 188283-188690 | AMW17944.1 | ATGCCACTAGTGCGTATCGACTTAACCTCTGACCGGCCACGCGATCAGCAACGCGCCATCGCCGACGCCGTGCATGACGCTCTGGTTGAAGTGTTGAAAATCCCTGTACGAGACCGATTTCAGATCATCACGGCTCATGAGGCCGCAGACATCATCGCAGAGGATGCGGGACTGCGCTTCGCGCGATCGTCCCGTGTGGTGATCGTGCATATCTTCACGCAGACCGGCCGTACCGTGGAGACCAAGCAGCGGATCTTCGCCGAGCTGGCCGACAGGCTGGCTGCCGTCGATGTGGCGGGCGCGGATCTTTTCGTGACGATCAGTGAAAATGGGCCGCAGGATTGGTCTTTCGGTTTCGGTAACGCGCAGTACGTTACCGGTGAGCTGGCTGTGCCCGCCGCAGCGAGC |
| *Mycobacterium chelonae* subsp. *bovis* QIA-37 (2) | CP010071.1 | 3591438-3591794 | AMW21227.1 | CGCCTGACCTCCACGGATTCCCCAATTCTCTGTCGCACTCTCACGCAGCACAATGGTGACGTGATCCGGCGGTATCCCCAGTAAGGAAAGCCGTTTCACAATCTCGGCATAGAGATTTCGTTTCGCCCCTATCGATCGGCCCGCGAAGCAGTCGATGGAAACAAATGTGTACAGCTCCGGCTGGGCCAGGTTTGGCGCGTGCGAAAACCTGTGGGGTTCATGCACGACAAGGCGTACATGCTTATCCCCTTCCGGTATCTGAAAGGCAGCGACCAGCGCGCCATGGACCGCATCGATGATCGCCACCTCTTCAGGCTCGGTGTATCGCCGACGGACCTCGACCACTGAACTGGGCAT |
| *Mycobacterium chubuense* NBB4 | CP003053.1 | 335970-336362 | WP_041782431.1 | CACGGTGACGTCGAAACCGAGATCGACATCGCAAGCGGCTTGCCCGCCGCGGATTCCCCAGTTCTCGAGGGCGCTTTCCCTCAGCACGATGGTCACGTGATCGGGCGGGATGCCCAGTGTGCTCAGCCGGTCCACGATCTGGCCGTAGAGATTGCGTTTGGCATGTATCGACCGACCCGCAAAGCAGTCGATCGCCACGAAGGTATGCAGCTCCGGCTTCGCCAACGTCGGTGAGTGTGAGAACCGGTGGGGTTCATGGGCGACCAGGCGGACATGCCTGTCGTTGAACGGAATCCCGAACGCGGACACGAGCGCCTGGTGAACCGCATCGATGATCGCCACCTCTTCGGCTGCGTCGTAGCGCCGTCGGACCTCGATGACCGTACTGGGCAT |
| *Mycobacterium colombiense* CECT 3035 | AFVW02000002.1 | 1110610-1110996 | EJO90307.1 | ATGCCGTTGTTGTATGTCGACCTCGTCGAGGGCCGTTCGGCGCCGGAGGTTCGTGCACTGCTGGACACGATTCACGACGCGGTGGTCGAGGCGTTCGGCGTCCCGGAGCGGGATCGCTACCAGGTGGTGCGCACCCATCCGGCCCACGAGATCGTCACGCTGGACACCGGTCTGGGCATCGACCGTTCGTCTCGCCAGGTCGTCCTGCACGTGGTGAGCCGGCGACGCCCGCGCGAGCTCAAGCAGAAGTTCTATGAGCTGCTGGCGTCGCGTCTCTCGGACCGCTGCGGACTCGATCCTGCCGACCTCATCGTCGCGGTCACCGAAAATGGCGACGAGGACTGGTCTTTCGGCCTCGGCAGGGCGCAGTTCCTCACCGGTGAGCTC |
| *Mycobacterium conceptionense* CCUG 50187 | LQOP01000021.1 | 54284-54676 | ORV24298.1 | GACGTCGAGCTCGAAGCCCAGGTCGATATCGCACGCGGCCTGCCCGCCCCGGATGCCCCAGTTCTCGAGCGGGCTCTCACGTAAGACGATCGTGACATGGTTGGCCGGAATGCCCAGTTTCTCCAGCCGAATGACGATCTGCCGGTAGAGGTTTCGCTTCGCCTCTACTGATCGACCGGAAAAGCAGTCGATTCCGACGAGCGTGTAGAGCTCGGGATGAGCCAACCCCGGCGAGTACGAGAACCGATGTGGCTGATGGGAAACCAGTCGGACATGCTTGTCGCCGACGGGTATCTGAAACGCCGCCACGAGCGCATCGTGCACCGCGTCGATTATCGCCACCTCTTCGGATTCGGTGTAGTGGCGCCTGACCTCGATGAGCGTGCTGGGCAT |
| *Mycobacterium conspicuum* DSM 44136 | LQOR01000047.1 | 87417-87803 | ORV38418.1 | CAGCGCTCCCGTCAGAAACTGTGCGCGCCCGTGGCCGAAAGACCAGTCCTCGTCGCCGTTTTCGGTGATCGATACGATCAGGTCTGCCGGGTCCGTCCCGCACCGTTCGGCGAGATTGGACGCGAGCAGTTCATAGAACTTTTCCTTCGCCTCGCGGGTGCGTCGCCGGCTCACCACGTGCACCACCACCAACCGCGATGAGCGCTCGATGCCCAGACCGGTATCCCAGGCGACGATCTCGTGGGCCGGATGGGTGCGCACCACCTGGTAGCGGTCGCGCAGCGGGACCCCGAACGCGTCGACCACCGCGTCGTGGATTGCGTCCAGCAGTTCCGTGACCTCGGCCGGCGTGCGACCCTCGATGAGGTCGATGTACAGCAGCGGCAT |
| *Mycobacterium cookii* DSM 43922 | JACKUP010000026.1 | 30849-31235 | MCV7331058.1 | ATGCCGCTGCTGTACGTCGACCTGATCGAGGGTCGCTCGCCCGCCGAGATCCGCACGCTGCTGGACGCCATTCACGACGCCGTCGTCGAGGCATTCGCGGTGCCGACGCGCGACCGCTATCAAGTGGTGCGCACCCATCGGGCCCACGAGATCGTGGTGTGGGACACCGGCCTTGGAATCGACCGGTCGTCTCAGCTGGTTGTGCTGCACGTGGTGAGTAGACGGCGCTCTACGGAGATGAAGCAACGGTTCTACGAGTTGGTGGCCGCCAACCTCGCCGCGCGGTGCGGGGTCGACTCGGCAGATCTGGTCGTGTCGGTCACCGAGAACGATGATGCTGACTGGTCGTTCGGCCATGGCCGGGCGCAGTTTCTGACGGGAGAGCTG |
| *Mycobacterium cosmeticum* DSM 44829 | CCBB010000001.1 | 10639-11040 | CDO05246.1 | ATGCCCTTGGTTCGCATCGACCTCGCCGACCACCACAGCCCCGAACAGATCCGCGCCATCGCCGACCGCGTTCATCAAGCCATCGTGGACGTGCTCGGCATTCCGGAGCGGGACCGCTTCCAGATCATCACCGCCCACCGGCCCGGCGAGATCGTGGCCCTGGACGCCGGTCTGGGCTTCGACCGCTCGGCCGATGTGGTGATGGTCCACATCTTCACGCAGGCGGGCCGCTCGGACACCACCAAACAGGAACTGTTCGCCCGGATCGCCGAGCGCCTCGCGGGGGCCGGTGTCGACGGGCGCGACATCTTCGTCGGGATCACCGAGAACGGGCCGCAGGACTGGTCGTTCGGCTTCGGCAAGGCCCAATATCTGACCGGGGAACTCGCCATCCCGTCGGCC |
| *Mycobacterium dioxanotrophicus* PH-06 | CP020809.1 | 1641494-1641883 | ART68497.1 | ATGCCCCTGGTCCATATCCACGTGATCGAATCGCGGCGCACCGCTGAGCAATTGAGACAGCTGGCGGACACCATCCAGGACGTGATGCTCGAACACTTCGCGGCACCACCTCGGGACAGATACCAGATCATCACCGAACACAAACCCGGGCACATCATCGCCGAGGACACCGGCTTGGGCTTCGAACGGACCGATGACGTCGTCGTCATCCAGATCGTCCAGCAGGGCCGTACCTCGGACCAGAAACAAGCCATGTATACGGCGCTGGCCGAACGTCTCAAAGTCGCATCAGCTTTGGCCCCAACGGATTTGATTGTCTCCGTCGTCGAAAACTCCAAAGAGGACTGGTCATTTGGGTACGGCATCGCGCAGTTCATTGCCGGACAGCTG |
| *Mycobacterium dioxanotrophicus* PH-06 (2) | CP020809.1 | 823686-824042 | ART73394.1 | GTGCCGAGCGCTCTGATCGAGGTCCGCAAGGGCTACACCGCAGCCGAAGAGGTGGCTCTCATCGATGCGGTGCATGCCGCGCTCGTCGCTGCCTTCCAGATTCCCGAGGAAGACAAGCACGTGCGGCTGGTATGCCACGAGCCGCATCGGTTCGCGTATTCACCGAAGCTCGCGGCTCCCGATCTGTACACGCTCGTCACCATCGACTGCTTCGTGGGCCGCTCGGTGCAGGCGAAGCGCAATCTCTACGGCGAGATCGTCAATCGCTTGGGTGCCTTTGGAATTCCTGCCGACCACATCACGATACTGCTGCGTGAGAGTGCGCTTGAGAACTGGGGTGTGCGGGGCGGTCAGGCA |
| *Mycobacterium europaeum* CSUP P1344 | CTEC01000002.1 | 547791-548177 | CQD16449.1 | CAGCTCCCCGGTGAGGAACTGCGCCCGGCCGTGGCCGAAAGACCAGTCCTCGTCGCCGTTTTCGGTGATCGACACGACCAGGTCGGACCCGTCGAGCCCGCACCGGTCGGCCAGGTTGACGGCCAGCAACTCGTAGAACTTCTCCTTCATCGCCCGCGTGCGCCGCCGGCTCACCACGTGCACAATCACCTGTTGCGACGAGCGCGCGATGCCGAGGCCGGTGTCCAAAGCGACGACCTCGTGGGCGGGATGGGTGCGCACCACTTGATAGCGGTCCCGCGCCGGCACCCCGAACGCTTCGACGACGGATTCGTGAACCGCGTCGAGCAGCACCCGGACCTGCGACGGCGTGCGACCCTCGATGAGGTCGATGTACAGCAGTGGCAT |
| *Mycobacterium farcinogenes* DSM 43637 | CCAY010000005.1 | 43843-44199 | WP_036389747.1 | ATGCCCAGCACGCTCATCGAGGTCAGGCGCCACTACACCGAATCCGAAGAGGTGGCGATAATCGACGCGGTGCACGATGCGCTCGTGGCGGCGTTTCAGATACCCGTCGGCGACAAGCATGTCCGACTGGTTTCCCATCAGCCACATCGGTTCTCGTACTCGCCGGGGTTGGCTCATCCCGAGCTCTACACGCTCGTCGGAATCGACTGCTTTTCCGGTCGATCAGTAGAGGCGAAGCGAAACCTCTACCGGCAGATCGTCATTCGGCTGGAGAAACTGGGCATTCCTGCCAACCATGTCACGATCGTCTTACGTGAGAGCCCGCTCGAGAACTGGGGCATCCGGGGCGGGCAGGCC |
| *Mycobacterium farcinogenes* DSM 43637 (2) | CCAY010000029.1 | 42138-42527 | WP_036390357.1 | TAGCAGACCCTCGATGAACTGCGCGCGCCCGTCACCAAAAGACCAGTCCGCGCGACTGTTCTCTACCATCGACACAATCAGATCCGCAGGTTTCAGGCCTGTTTGTTCGCGGAGCCGTTGCGCCAGAGCTCGGTACGTTTCTTGTTTCTGATCAGATGTGCGACCTTGCTGGAAGACGTGCACGAGGACAATGTCGTCGGTCCGGTCGAATCCGAGCCCTGTGTCTTCGGCGATGATCTGGCCCGGTTTGTGCTCGGTGATGATCTGGTACCTGTCGCGTTCAGGAGCGGCGAAATACTCAAGCATGACGTCTTGGATTGTGTCGGCGACCTGACGCAGCTGAGCCGGTGTGCGCCGGCCCTCGATGACATGAATATGTACGAGTGGCAT |
| *Mycobacterium florentinum* DSM 44852 | LQOV01000006.1 | 46922-47308 | ORV55690.1 | CAGTTCTCCGGTGAGGAACTGCGCTCGGCCGTGACCGAAAGACCAATCCTCGTCCCCGTTTTCGGTGATCGACACGATCAGATCGGCCGGGTCGACCCCGCATCGCTCGGCGAGATTGGCAGCCAGCAACTCGTAGAAGCGTTCCTTCATGGCGCGGGTGCGTTGGCGACTGACCACGTGCACGATCACCAACCGCGCTGAGCGGTCGATACCGAGACCGGTGTCCCAGGCCACGATCTCGTGCGCCGGATGGGTGCGCACCACCTGGTAGCGGTCGCCCGGCGGGACCGCGAACGCGCCGAGGACGGCGTCATGCGCGGCGTCCAGCAACGCCTGGAGCTCCGATGGGGTGCGACCCTCAATCAGATCGAAGTACAGCAGCGGCAT |
| *Mycobacterium fluoranthenivorans* DSM 44556 | JAANOW010000001.1 | 748506-748898 | NIH93774.1 | GTGCCCAGCTCACTGGTCGAAGTCCGCAGGCAGTACACCGTGGCGGAGGAGATCGCCATCATCGACGCCGTCCATGACGCCCTGGTGGCGGCATTCCGGATTCCGTTGGCGGACAAACATGTACGGCTGGTGGTGCACGCGCCGCACAGATTCGCCCACTCGCCGGAGCTGGCCGATCCGCAGCGCTACACCCTGGTCACGATCGACTGTTTCGCCGGGAGATCGGTCCAGGCCAAGCGGGGGTTGTTCGGCGAGATCGTCAGTCGACTGGGCGGCCTGGGTATCCCGGCCGACCATGTGACGATCGTGCTGCGCGAGAGTGCCCTGCAGAACTGGGGGATCCGCGGCGGTCAGGCGGCCTGCGATGTCGACCTCGGGTTCGAGGTGACCGTC |
| *Mycobacterium fortuitum* subsp. *fortuitum* DSM 46621 | ALQB01000054.1 | 23924-24316 | EJZ13450.1 | ATGCCCAGCACTCTCATCGAAGTACGACGGACTTGCGGCGAGGCCGACGAGGTGGCGATCATCGATGCGGTGCACGATGCTCTGGTGGTCGCGTTCCGCATTCCCGCCGGCGACAAACACGTTCGGCTGCTATCCCATGAACCTCACAGGTTCTCGTGTTCTCCGGAGCTTGCGCAGCCCGAGCTCTACACCCTCGTGACGGTGGACTGTTTTTCTGGTCGATCCGTGCAGGCCAAGCGAAACCTGTACCGGGAAATCGTCAGCCGCCTTCAGGGCTTGGGCATCCCGCCGAACCACGTGACCATCCTGCTGCGGGACCATCCGTTGCAGAACTGGGGCGTTCGCGGCGGGCAGGCCGCCTGCGATGTCGACCTGGGTTTCGACGTCAACGTG |
| *Mycobacterium franklinii* DSM 45524 | MVHQ01000038.1 | 18480-18887 | ORA54667.1 | ATGCCACTAGTACGTATCGAGCTGACCTCGGACCGGTCCCGTGCGGAGCAACGCGCCATTGCCGACGCCGTGCACGACGCGTTGGTGGCGGTGTTGAAAATCCCTGCGCGAGACCGATTCCAGATCATCACCGCACACGATGCGGCGGACATCATCGCTGAAGACGCCGGGCTGGGATTTCAGCGGTCGGGCCGGGTGGTGATCGTGCACATCTTCACTCAAGCCGGCAGGTCCACCGAGACCAAGCAGAGGATTTTCGCCGAGGTGGCCAAGAGGCTTGCCGTCGTCGATGTGGACGGCGCCGATCTGTTCGTGGCAATCAGCGAAAACGGGCCGCAGGATTGGTCTTTCGGCTTCGGGGCGGCGCAGTACATCACCGGCGAGCTGGCCGTGCCTGCCGCCGCGAGC |
| *Mycobacterium goodii* CCUG 58730 | JACKUB010000026.1 | 300176-300571 | MCV7294147.1 | CGGTACGGCGAGTTCGCCGGTGACGTACTGGGCCTTTCCGAAACCGAACGACCAGTCCTCGGGTCCGTTCTCTGCGATGCCGACGAAAATGTCGTCGCCGCGGACTCCCACCGCCTCCAGCTGCGCCGCGATCTCGGCGTAGAGCCGTTCCTTCGTGTCGATGGAACGCCCGCGCTGCGTGAAGATCTGGATGATCACGGTGTCCGGCGAGCGGTCGAACCCGAGGCCGGCGTCCAGCGCGATGATCTCTCCGGGGCGATGCGGGGTGATGACCTGGAACCGGTCACGCTCGGGGATTCCGAGTACCGCGACCAGGCTTCGGTGGATGGCGTCGGCGACGTTTCGGATGTGTTCATTTCCATGGTGGTTCAGCAGATCGATACGAACCAGTGGCAT |
| *Mycobacterium gordonae* CTRI 14-8773 | LKTM01000380.1 | 12232-12639 | WP_055581702.1 | ATGCCACTGCTGTACATCGACCTCATCGAGGGCCGCACACCGTCGGAGGTGTGCTCGCTGCTCGATGCCGTACACGACGCGGTCGTCGAAGCGTTCGGGGTGCCCCCGCGCGACCGCTATCAGGTGGTGCGCACCCATCCGGCGCACGAAATCATCGCGCTGGATACGGGCCTCGGCATCGACCGCAGCCCGTCGCTGGTAATCGTGCAGATGGTGAGCCGGCGCCGCCCACCGGAGCTCAAGCAGAAGTTCTACGACCTACTGGCGTCGAACCTCGCCTTGCGATGCGGACTGGACCCAGCCGACCTGATCGTCTCGGTCACCGAAAACCAGGACGAGGATTGGTCTTTCGGCCACGGCCGGGCGCAGTTCGTCACCGGGGAACTCGCAGCGGAGGCACCACGGTGA |
| *Mycobacterium heraklionense* JCM 30995 | CP080997.1 | 1718456-1718848 | QZA09194.1 | ATGCCCACTGTCCTGATCGAGGTCCGCCGCCGCTACGAGCCGGCCGAGGAAGTCGCGATCATCGATGCCGTCCACGGCGCGCTGGTGACCGCCTTCCAGACCCCGGCCACGGACAAGAACGTGCGGTTGGTCGTCCATGAGCCGCACCGGTTCGCGGTTCCGGAGCAGCTCGCAAAGCCCGAGTGCCGCACGCTGATCTCGATCGACTGCTTCTCCGGCCGGTCCCTGGAGGCCAAGCGGCAGCTGTATGCCGGGATCGTCGAAAACCTTGCCGCGCTGGGCATTCCCGCTGACCACGTGATGATCACCCTGCACGAGGTGGATCGGGACAACTGGGGCATCCGGGGCGGGCAGGCCGCATCCGACGTCGACCTGGGGCTCAACGTCCGGGTG |
| *Mycobacterium hiberniae* DSM 44241 | JACKSF010000293.1 | 145488-145844 | MCV7085505.1 | GTGCCCACCGTCCTGATCGAGGTCCGCCGCCGCTACGAGCCGGCCGAAGAAGTTGCGATCATCGACGCCGTCCACGGCGCGCTCGTGACGGCCTTCCAGATCCCGGCCGCCGACAAGAACGTGCGGCTGGTCGTGCACGAGCCGCACCGGTTTGCGGTTCCGGCGCAGCTCACCAACCCCGAGTACCGCACTCTGGTCTCCATTGACTGTTTCTCCGGCCGGTCGCTTGAGGCCAAGCGGCGGCTTTATGCCGGGATCGTCGAAAACCTTGCTGCGCTGGGTATTCCCAGTGAACATGTGATGATCACCCTGCACGAAGTGGATCGAGAGAACTGGGGTATCCGGGGCGGGCAGGCC |
| *Mycobacterium icosiumassiliensis* 8WA6 | FJVP01000024.1 | 42405-42761 | WP_067974581.1 | GGCCTGCCCGCCTCGAATGCCCCAATTGTCGCGATCCACTTCGTGCAGGGTGATCATCACATGGTCAGCGGGAATGCCCAGCGCAGTAAGGTTTTGGACGATACCGGCATAAAGCAGCCGCTTGGCCTCAAGTGACCGGCCCGAGAAGCAGTCAATCGATACCAGGGTGCGGTAGTCGGGCCTGGCGAGCTGCGCCGGGACCGCAAACCGGTGCGGCTCGTGGACGACCAACCGCACGTTTTTGTCCTTGGCCGGGATCTGGAAAGCGGTCACGAGCGCGCCGTGGACGGCATCGATGATCGCGACTTCCTCGGCCGGTTCGTAGCGGCGGCGAACCTCGATCAGGACGGTGGGCAT |
| *Mycobacterium immunogenum* DSM 45595 | JACKUV010000009.1 | 175031-175438 | MCV7303835.1 | TGCACTCGCGGCGGGGACCGCCAGCTCACCGGTGACGTACTGGGCTCTACCGAAGCCGAAAGACCAATCCTGTGCCCTGTTTTCGCTGATCGCGAGGAACAGATCGGCGCCATCCACCTCGACGGCGGCAAGTCTGTCCGCGAGTTCGGCGAAGATCCGCTGTTTGGTCTGCGTTGTGCGCCCGGCCTGGGTGAAGATGTGCACGATCACCACGCTGCCTGATCGCTCAAACCCCAACCCCGCGTCTTCGGCGATGATGTCGGCGGCATCATGTGCCGTGATGATCTGAAATCGGTCGCGTGCCGGGATTTTCAAGACCGCGACCAGAGCGTCGTGCACGGCGTCCGCGATAGCGCGCCGCTGCGCCAGGGACCGGTCCGCGGTGAGGTCGATGCGTACCAGTGGCAT |
| *Mycobacterium interjectum* DSM 44064 | LQPB01000059.1 | 58751-59146 | ORV85659.1 | GGGTCATGTCAGCTCCCCGGTGAGGAACTGCGCCCGACCGTGCCCGAAAGACCAGTCCTCATCCCCATTTTCGGTGATCGAGACGATCAGATCGGCCGGGTCGATCCCGCACCTCTCGGACAGGCTGAGCGCCAGCAGCTCGTAGAACTTCTCCTTCATCGCGCGGGTGCGCCGCCGGCTCACCACGTGCACGATCACCTGCTGCGCCGACCGGGCGATGCCGAGACCGGTGTCCCAGGCGACGATCTCGTGGGCGGGATGGGTGCGCACCACCTGGTAGCGGTCGCGCGGCGGCACGCCGAACGCCTCGACCACGGCCTGGTGGACGGCGTCCAACAGCGCCCGAACCTCCGCCGGCGTGCGGCCCTCGATGAGGTCGATATACAGCAACGGCAT |
| *Mycobacterium intracellulare* subsp. *intracellulare* ATCC 13950 | ABIN01000192.1 | 8216-8602 | WP_009955983.1 | ATGCCGCTGTTGTACATCGACCTGATCGGGGGCCGCTCTCCGTCGGAGGTTCGGGCGCTGCTGGACGCGATCCACGAGACGGTGGTCGAAGCGTTCGGTGTTCCCGAGCGTGATCGCTATCAGGTGGTCCGCACTCATCCGGCCCACGAGGTCATCGCGCTGGACACCGGTCTCGGCATCGACCGCTCGGCGCGGCAGGTCATCCTGCACGTGGTCAGCAGGCGACGTCCGCGCGAACTCAAGCAGAAGTTCTACGAACTGCTGGCGTCCCGCCTCGCCGACCGGTGCGGACTCGACCCGGCGGACTTGATCGTCTCGGTCACCGAAAACAACGATGAGGACTGGTCTTTCGGCCACGGCCGCGCACAGTTCCTCACCGGTGAGCTG |
| *Mycobacterium intracellulare* subsp. *chimaera* DSM 44623 | LQOO01000073.1 | 187937-188323 | ORV23538.1 | CAGCTCACCGGTGAGGAACTGTGCGCGGCCGTGGCCGAAAGACCAGTTCTCATCGTTGTTTTCGGTGACCGAGACGATCAAGTCCGCCGGGTCGAGTCCGCACCGGCCGGCGAGGCGGGACGCCAGCAGTTCGTAGAACTTCTGCTTGAGTTCGCGCGGACGTCGCCTGCTGACCACGTGCAGGATGACCTGCCGCGCCGAGCGGTCGATGCCGAGACCGGTGTCCAGCGCGATGACCTCGTGGGCCGGATGAGTGCGGACCACCTGATAGCGATCACGCTCGGGAACACCGAACGCTTCGACCACGGTCTCGTGGATCGCGTCCAGCAGCGCCCGAACCTCCGACGGCGAGCGGCCCTCGATCAGGTCGATGTACAACAGCGGCAT |
| *Mycobacterium intracellulare* subsp. *yongonense* 05-1390 | CP003347.1 | 2588339-2588725 | AGP64006.1 | ATGCCGCTGTTGTACATCGACCTGATCGAGGGCCGCTCGCCGTCGGAGGTTCGCGCGCTGCTGGACGCGATCCACGAGACCGTGGTCGAAGCGTTCGGTGTTCCCGAGCGTGATCGCTATCAGGTGGTCCGCACTCATCCGGCCCACGAGGTCATCGCGCTGGACACCGGTCTAGGCATCGACCGCTCGGCGCGGCAGGTCATCCTGCACGTGGTCAGCAGGCGACGTCCGCGCGAACTCAAGCAGAAGTTCTACGAACTGCTGGCGTCCCGCCTCGCCGACCGGTGCGGACTCGACCCGGCGGACTTGATCGTCTCGGTCACCGAAAACAACGATGAGGACTGGTCTTTCGGCCACGGCCGCGCACAATTCCTCACCGGTGAGCTG |
| *Mycobacterium koreense* KCTC 19819 | NCXO01000011.1 | 47649-48041 | OSC34371.1 | GATCGTGACCGGATATTCCAGGTCGACGTCGCAGGCCGCCTGACCACCCCGGATCCCGAAATTCTCGGTGGTGTTCTCCCGCACCACGATTGCGACGTGATCGGCGGGGATGCCCAGCGCGCCAAGGCGTTCGACGATGCCGCGATAGAGGTTGCGTTTGGCCTGCACCGAGCGGCCGGCGAAACAGTCGATCGTCACCAGCGTGTAGCGCTCCGGGTCGGTGAGGTTCGGCGCGGTGGCGAACCGGTGCGGTTCATGCACGATCAGGCGCAGGCAACGGTTGTCGGCCGGGATCTCGAACGCGTCGACCAGCGCGTCGTGCACGGCGTCGATGAGGGCGATCTCGTCGTCTCGGCTGTAGGTGCGGCGGACCTCGATCACGGTGTTCGGCAT |
| *Mycobacterium kubicae* JCM 13573 | BLKU01000003.1 | 1522811-1523215 | GFG65164.1 | GCAAGGCGGCACTCACTTGAGCTCTCCGGTGAGAAATTGCGCCCGGCCATGTCCGAACGACCAATCATCGTCGTCGTTTTCGGTGACCGAGATGATGAGATCGGCCGGGTCGATGCCGCATCGCTCGGCGAGATTGCCTGCAACGAGTTTGTAGAACCGCTCTTTGAGCTCTTTGGGCCGGCGCCTGCTCACCACGTGCAAAACGACTAGCCGAGCAGAGCGGTCTATACCCAGCCCGGTGTCCAGCGCAACGATTTCGTGAGCCGCATGGGTGCGCACCACCTGATAGCGGTCGCGCTGCGGCACTCCGAATGCTTCGACGACGGCATTGTGAATCGCGTCCAACAACCCACGCACCTCAGCCGGCTTCCGGCCCTCGATCAAGTCCACATACAGCAGCGGCAT |
| *Mycobacterium kumamotonensis* DSM 45093 | MVHU01000013.1 | 77510-77866 | ORA79922.1 | GTGCCCACCGTCCTGATCGAGGTCCGCCGCCGCTACCAGCCGGCCGAAGAAATCGCGATCATCGATGCCGTCCACGCCGCACTGGTGACCGCCTTCCAGATCCCGGCCAAGGACAAGAACGTGCGGCTGGTCGTGCATGAACCGCACCGGTTCGCGGTTCCTGAGCACCTGGTGCAGCCGGAGTACCGCACCCTGATCTCGATCGACTGCTTCACCGGCCGGTCCCTGGACGCCAAGCGGACCCTCTACGCCGAAATCGTGGAACGCCTTGCCGCATTGGGTATTCCGCGTGACCACGTGATGATCACCCTGCACGAGTTCGACCGGCAGAACTGGGGAATCCGCGGCGGCCAGGCC |
| *Mycobacterium kyogaense* NCTC 11659 | QJUA01000030.1 | 46574-46930 | WP_111511332.1 | ATGCCCAGCACTGTCATTGAAGTTCGTCGGCGTTATCGCGTTGATGAGGAGATCGCGATCATCGATGCCGTTCACGATGCACTGGTCGCCGCGTTCGAGATACCGGCCGCTGACAAGCACGTCCGGCTCGTCAGCCATGAACCGCATCGATTCTCACACTCTCCGGGATTGGCCCAGCCGGAGTTGTACACGTTCGTAGCCGTCGACTGTTTCGCAGGACGCTCGGTGGAAGCGAAACGGAACCTGTATCGGGAGACCGTGGACAGGCTGGGCGCTCTAGGCATCCCAGCCGATCACGTGACCATCGTCTTGCGTGAAAGCGCCGTCGAGAATTGGGGCGTCCGTCGCGGGCAGGCA |
| *Mycobacterium llatzerense* MG13 | LXOV01000012.1 | 257722-258114 | MCT7364289.1 | GACATTCACGTCGAACCCGAGGTTGACGTCGCAGGCCGCCCGCCCACCGCGGATGCCCCAGTTCTCGAGCGCACTCTCGCGGAGCAGGATCGTGATGTGATCGGCCGGAATGTCGAAGGCGGCCAGGCGGCCGACGATCTCCCGGTACAGCTCCCGCTTGGCATCCACCGATCGGCCGGCGAAACAGTCGATGGTCACCAGCGTGTACCGCTCGGGGTGTTCGAGGTGCGGGGCATGCGAAACCCGGTGCGGTTCATGGACCAGCAGCCGGACGTGCTTGTCGCCGACCGGGATCCGGAACGCGGCAACCAGCGCACCGTGGACGGCATCGATGATGGCGACCTCGTCGGGCTGGCTGTACTGGCGGCGGACCTCGATCAGTGAACTCGGCAT |
| *Mycobacterium lutetiense* DSM 46713 | JAGIOP010000002.1 | 352108-352464 | MBP2452290.1 | ATGCCCAGCACGGTCATCGAGATAAGGCGGCCTTGCCGTCGGTCCGACGAGGTGGCGATCATTGACGCGGTGCACGAAGCACTCGTGGTCGCTTTCCAGATCCCGGCCGGCGACAAGCATGTCCGGCTGGTATCCCATGAGCCGCATCGTTTCTCGCACTCGCCGGGCCTGGCGCAACCTGAGCTGTACACCCTCGTGACGATCGACTGCTTTTCCGGGCGATCGGTGCAGGCCAAGCGCAGCCTCTATCAAGAGATTGTCGGTCGACTGGAAGCGCTGGGCATCCCACCCGACCACGTCACCATCGTGCTGCGCGACCACCCCATGCAGAACTGGGGAATTCGTGGTGGGCAGGCC |
| *Mycobacterium mageritense* strain CIP 104973 | CCBF010000004.1 | 501989-502384 | CDO26662.1 | CGGAACCGCGAGTTCGCCGGTGAGGTATTGGGCCTTGCCGAAGCCGAAAGACCAGTCTTCGGGCCCGTTCTCGACGAGGCCGACGAACATGTCGTTGCCCCGCACTCCCACGGTCTGCAGTTGCGCAGCCAACTCGGCGAACAGGCGCTCCTTCGTCTCGATCGAACGGCCGCGCTGCGTGAAGATCTGGACGACGACGACATCGCGGGAGCGGTCGAAATCCAGCCCGGCGTCCAAGGCGATGATCTCGTCTGCGGCGTGGGTGGTGATGACCTGGAATCGGTCGCGCTCGGGGATTCCGAGGACCGTGACCACGCCACGATGGATGGCATCGGCGATGTCGCGGATGTGGTCGCGCTCGTGATGGTTCAGCAGATCGATGCGGACCAGTGGCAT |
| *Mycobacterium mantenii* DSM 45255 | MVHW01000001.1 | 312565-312954 | ORB09275.1 | ATGCCGCTGCTGTATATCGACCTCATCGAGGGTCGTACGGCGTCCGAGGTTCGTGCACTGCTGGACGCGATCCACGAGACGGTGGTCGAGGCGTTCGGCGTCCCGGAGCGGGATCGCTACCAGGTGGTGCACACTCACCCGGCGCACGAGATCGTTACGCTGGATACCGGTCTCGGCATCGATCGTTCGTCACGCCAGGTGGTCATGCACGTGGTGAGCCGACGGCGTCGCCGTGAGCTCAAGCAGAAGTTCTATGAGTTGCTGGCGTCCCGTCTCGCCGACCGCTGCGGCCTGGACCCTGCCGATCTGATCGTCTCGATCACCGAAAACGATGATGAGGATTGGTCTTTCGGCCATGGTAGGGCGCAGTTCTTGACCGGTGAGCTCACG |
| *Mycobacterium minnesotensis* DSM 45633 | JACKRY010000412.1 | 180565-180921 | WP_083022268.1 | ATGCCCACTGTCTTGATCGAAGTTCGCCGCCACTACTCGCAGGCCGAGGAAGTCGCGATCATCGACGCCGTCCACAGCGCTCTGGTGACCGCTTTCCAGATACCGGCAAAGGACAAGAACGTCCGGTTGGTGGTGCATGAGCCGCACCGCTTCGCGGTGCCCGCACAGCTCGCCCAGCCCGAGTTCCGGACCTTGGTCTCCATCGACTGTTTCTCCGGCCGGTCAGTGGAGGCCAAGCGACTGCTCTACGCCGGCATCGTTGAAAATCTTGCAGAACTTGGTATTCCAGGCGACCACGTGATGATCACCCTGCACGAGTCGGATCGCGACAACTGGGGTATCCGGGGCGGGCAGGCG |
| *Mycobacterium mucogenicum* DSM 44124 | CYSI01000007.1 | 5973713-5974105 | KAB7751185.1 | GACATTCACATCGAACCCGAGGGCGACGTCGCAGGCGGCCTGTCCGCCGCGGATACCCCAGTTCTCGAGCGCGCTCTCGCGGAGCAGGATCGTGATGTGGTCGGCCGGTATGTCGAAGGCGGCAAGGCGGCCCGTGATCTCGCGGTACAGCGCACGCTTGGCGTCCACCGACCGCCCGGCGAAACAGTCGACGGTCACCAAGGTGTACCGCTCGGGATGCGCGAGATGCGGCGCATGCGAAAACCGGTGCGGCTCATGGACCAGGAGCCGGACGTGCTTGTCGCCGACCGGGATCTGGAACGCGGCGACCAGTGCAGCGTGGACGGCATCGATGATGGCGACCTCGTCGGACTGGCTGTACTGACGTCGGACCTCGATCAGTGAACTCGGCAT |
| *Mycobacterium nebraskense* DSM 44803 | LQPH01000126.1 | 33375-33761 | ORW21702.1 | CAGCTCCCCGGTGAGAAACTGCGCCCGGCCGAGGCCGAAAGACCAATCCTCGTCCCCGTTTTCGCTGATCGAGACGATCAGGTCGGCCGGGTCGAGCCCGCACCGGTCGGCCAGGTTGCCGGCCATCAGCTCGTAGAACTTCTCCTTCATCGCCCGCGTGCGCCGCCGGCTCACCATGTGCACGATCACCTGTTGCGGCGAGCGGGTGATGCCGAGACCGGTATCCAGAGCGACGATTTCGTGGGCGGGGTGGGTGCGCACCACCTGATAGCGGTCCCGCGGCGGCACCCCGAACGCTTCGACCACGGACTCGTGAATCGCGTCGAGCAACGCCCCGACCTCCGACGGCGTCCGGCCCTCGATGAGGTCGATGTAAAGCAGCGGCAT |
| *Mycobacterium neworleansense* ATCC 49404 | CWKH01000001.1 | 1199729-1200085 | CRZ14302.1 | ATGCCCAGCACGGTCATTGAGGTCAGGCACCGGTACACCGAACCTGAAGAGGTGGCGATCATCGACGCGGTGCACGATGCGCTGGTGGCCGCGTTCCAGATACCGCCCGGGGACAAGCATGTCCGGCTGGTGTGCCATGAGCCTCACCGGTTCTCACATTCGCCGGGTCTGGCGAGGCCTGAACTGTACACGTTCGTAGCGGTCGACTGCTTTGCCGGGCGCACTGTGGGGGCCAAGCGCACTCTCTACCGAGAGGTCGTCAATCGCCTAGAGGGACTTGGCATCCCGCCTGATCACGTCACGATCGTCTTGCGGGAAAGCGCTGTCGAGAACTGGGGAATTCGTGGTGGGCAGGCG |
| *Mycobacterium nivoides* DL90 | CP034072.1 | 6419774-6420130 | WP_124714003.1 | GGCCTGTCCGCCACGAACGCCCCAGTTCTCCAGCGCGCTCTCCCGCAGCACGATCGTGATGTGGTCGGCTGGGATGCCGACAGCCTCCAGGCGAGTGACGATCTCCCGGTAGAGATTTCGTTTGGCCGGTACCGATCGGCCGGCAAAGCAGTCGATGGTCACAAGCGTGTACAGCTCAGGCTTTGCCAGGCCCGGCGAATACGAGAGCCGGTGTGGTTCATGGGATACCAACCGGACATGCTTGTCGCCGGGAGGTATCTGGAATGCGGCCACCAGCGCATCGTGCACCGCGTCGATGATCGCGACCTCTTCCGATTCGGTGTACCGCCGTCTGACCTCGATGAGCGTGCTGGGCAT |
| *Mycobacterium nonchromogenicus* DSM 44164 | LQPI01000072.1 | 76160-76516 | ORW16595.1 | GTGCCCACCGTCTTGATCGAGGTCCGCCGCCGCTACGAGCCGTCCGAAGAGGTTGCGATCATCGACGCCGTCCACGGCGCGCTCGTGACGGCCTTCCAGATCCCGGCCGCCGATAAGAACGTGCGGCTGGTGGTGCACGAGCCGCACCGGTTTGCGGTTCCGGCGCAGCTCACCCAGCCCGAGTACCTCACCCTGGTCTCCATTGACTGCTTCTCGGGCCGGTCGCTCGAGGCCAAGCGGCGGCTTTATGCCGGGATCGTCGAAAACCTTGCTGCGCTGGGTATTCCCCGTGACCATGTGATGGTCACCCTGCACGAAGTGGATCGGGAAAACTGGGGTATCCGGGGCGGGCAGGCC |
| *Mycobacterium paraense* IEC26 | LQPM01000010.1 | 242709-243116 | ORW46045.1 | ATGCCATTGCTGTATATCGACCTCATCGAGGGCCGCACGCCGGCGGAGGTTCGGGCGCTGCTGGACGCCGTCCACGAGGCCGTGGTGGAGGCGTTCGGCGTGCCGCCGCGCGACCGCTACCAGGTGGTGCGCACCCATCCCGCCCACGAGATCGTCACCTGGGACACCGGTCTCGGCATCACCCGGTCGGCCCGGCAGGTGATCGTGCACATGGTGAGCCGGCGGCGCACCCGCGCGATGAAGGAGAAGTTCTACGAGCTGCTGGCGCTGAGCCTGTCCGAGAAGTGCGGGATCGACCCGGCCGATCTGATCGTCTCGATCACCGAAAACGGTGACGAGGATTGGTCATTCGGTAACGGCCGGGCGCAGTTTCTCACCGGGGAGCTGACATGACCCTGGGCACCCGCC |
| *Mycobacterium paraffinicum* M11 | MPNT01000007.1 | 50677-51069 | WP_073874042.1 | ATGCCGCTGCTGTACATCGACCTCGTCGAGGGCCGCACGCCGTCGGAGGTGCGCGCGTTGCTCGACGCCGTGCACGAGTCGGTCGTCGAGGCGTTCGGGGTGCCACCGCGCGACCGTTACCAGGTGGTGCGCAGCCATCCCGCCCACGAAATCATCGCGCTGGACACGGGCCTTGGCATCGCGCGGTCGGCGCAACAGGTGATCGTCCACGTGGTGAGCCGCCGGCGCACGCGGGCGATGAAGGAGAAGTTCTACGAACTGCTGGCCACCAACCTGGCGGAGCGGTGCGGGCTCGACCCGGCTGACCTGATCGTCTCGATCACCGAAAACGGCGACGAGGATTGGTCGTTCGGCCACGGCCGGGCGCAGTTTCTCACCGGGGAGCTCAAATGA |
| *Mycobacterium paragordonae* JCM 18565 | BLKX01000001.1 | 2993186-2993584 | GFG79472.1 | CGCCTCCGCGGTCAGTTCTCCGGTGAGGAACTGCGCCCGGCCGTGGCCGAAAGACCAGTCCTCATCTTCATTCTCGGTGATCGAGACGATCAGATCGGCCGGGTCGAGTCCGCATCGCTCGGCGAGCGTCGACGCCAGTAGCTCGTAGAACTTCTGCTTGAGTTCGCGCGGACGGCGTCGGCTCACCATCTGAACGATCACCAGTGACGGAGTGCGGTCGATGCCGAGACCCGTGTCCAGCGCGATGGTCTCGTGGGCCGGATGGCTGCGCACCACCTGGTAGCGGTCGCGCGGGGGTACCCCGAACGCATCGACAACCGCGTCGTGAACGGCGTCGAGCAGCGACCGGACCTCAGCCGGTGTGCGGCCCTCGATGAGGTCGATGTACAGCAGTGGCAT |
| *Mycobacterium parakoreensis* DSM 45575 | JACKUQ010000025.1 | 80013-80405 | MCV7315987.1 | ATGCCCAACACCGTGATCGAGGTCCGCCGTACCTACAGCCGCGACGACGAGATCGCCCTCATCGACGCCGTGCACGACGCGCTGGTCGACGCGTTCGCGATCCCGGCCGACAACCGCTGCCTGCGCCTGATCGTCCACGAGCCGCACCGGTTCGCCGCCGCGCCGAACCTGGCCGACCCCGAGCGCTACACCCTGGTGACCATCGACTGTTTCGCCGGCCGTTCGGTGCAGGCCAAACGCAACCTGTACCGCGGCATCGTCGAACGGCTTGGTGCGCTGGGGATTCCCGGTGACCACATCGCAATCGTGGTGCGGGAGAACACGACCGAGAACTTCGGGATCCGCGGCGGGCAGGCCGCCTGCGACGTCGACCTGGGGTATCCGGTCACCGTC |
| *Mycobacterium paraseoulense* DSM 45000 | MVIE01000021.1 | 81912-82298 | ORB38813.1 | CAGCTCCCCGGTGAGAAACTGTGCCCGCCCGTGGCCGAAAGACCAGTCCTCGTCACCGTTTTCGGTAACCGAGACGATCAGGTCGGCCGGGTCGAGCCCGCACCGCCCGGCGAGGTTGCCGGCCAGCAGTTCGTAGAGCTTCTCCTTCATCGCCCGCGCGCGCCGCCGGCTCACCACGTGCACGATCACCTGGTGAGACGAGCGGGTGATGCCGAGGCCGGTGTCCAAAGCGACGATCTCGTGGACGGGATGGGTCCGCACCACCTGATAGCGGTCACGCGGGGGCACGCCGAACGCTTCGACGACGGACGCGTGAACCGCGTCGAGCAACGCGCTGACCTCCGACGGCGTGCGGCCCTCGATGAGGTCGATGTACAGCAGCGGCAT |
| *Mycobacterium peregrinum* DSM 43271 | LQPP01000010.1 | 27418-27774 | ORW62730.1 | GGCCTGTCCGCCACGAATGCCCCAGTTCTGCAGCGGGCTGTCGCGCACGAGGATGGTGACGTGGTTCGGTGGGATGCCCAGCGCCTCCAGACGATTGACGATCTGCCGGTAGAGGTCTCGCTTGGCCTCGACCGACCGACCAGAAAAACAGTCGATCGTCACCAGGGTGTACAGCTCCGGTTGCGCCAGGCCCGGTGAGTGCGAGAACCGATGCGGCTCATGGGATACCAGCCGGACATGCTTGTCGTCGGCCGGGATGTGAAAGGCGGCCACAAGTGCGTCGTGCACGGCGTCGATGATCGCCACCTCTTCGGACTGGCGGTAGGGCCGCCGTACCTCGATGAGTGTGCTGGGCAT |
| *Mycobacterium phlei* DSM 43239 | LR134347.1 | 156101-156508 | VEG14313.1 | ATGCCACTAGTGCGTATCGACGTTACTTCCGACCGGTCACGTGATCAGCAACGCGCCATCGCCGACGCCGTGCATGACGCCTTGGTCGAAGTGTTGAAAATCCCTGCACGAGACCGATTTCAGATCATCACGGGGCATGAGGCCGCCGACATCATCGCAGAGGATGCGGGACTGGGGTTCACGCGATCGGCCCGTGTGGTGATCGTGCACATCTTCACCCAGGCCGGCCGTACCGCGGAGACCAAGCAGCGGATCTTCGCCGAGCTGGCCAACAGGCTGGCCGCCGTCGATGTGGCGGGCGCGGATCTTTTCGTGGCGATCAGCGAAAATGGGCCGCAAGATTGGTCTTTCGGCTTCGGTAACGCGCAGTACGTTACCGGTGAGCTGGCTGTGCCCGCCGCGGCGAGC |
| *Mycobacterium phocaicum* DSM 45104 | POTM01000034.1 | 20188-20580 | TLH64800.1 | ATGCCGAGTTCCCTGATCGAGGTCCGACGCCGGTACAGCCAGTCTGACGAGGTCGCGCTCATCGACGCCGTCCACGGTGCGCTGGTAGCCGCGTTCCGGATCCCGGTCGGCGACAAACACGTCCGGCTGGTGGCCCATGAGCCGCACCGGGTTTCGCACGCGCCGCATCTCGCGCACCCCGAGCGGTACACCCTGGTGACCATCGACTGTTTCGCCGGCCGGTCGGTGGCTGCCAAGCGCGAGCTGTACCGACAGATCGTGCGCGGCCTGGCCGCCTTCGACATTCCGGCCGACCACATCACGATCCTGCTGCGCGAGAGCGCGCTGGAGAACTGGGGCATCCGGGGCGGGCAGGCCGCCTGCGACGTCGGCCTCGGGTTCGATGTGAATGTC |
| *Mycobacterium phocaicum* DSM 45104 (2) | POTM01000047.1 | 287073-287459 | TLH67679.1 | ATGCCATTGCTCAACATCACCGTGATCCGGGGCCGCACCCCCGAAGAACTCCGTCAGCTGCTGGACAGCGCCCACCGGGCTGTTGTGGAGGCGTTCGGCATCCCCGAACGGGACCGCTACCAGGTCGTCGACGTCCGCGAGCCCGATGACGTTGTCGCGCAAGACACTGGACTGGGATTCGAACGATCAGACCAGCTAGTGATCATCCAGGTGGTCAGCCGCCGCCGCACCGCAGAGATGAAGCAGCGGTTCTACGAGCTGCTGGCCGAGCACCTGCATAGGGACTGCGGTTTGGACGCCAAAGACCTGATCGTGTCGATCACCGAAAACGGCGACGCCGATTGGTCGTTCGGCGCGGGCCGGGCACAGTTTGTCACCGGCGAACTC |
| *Mycobacterium porcinum* HMC1 | MSTD01000033.1 | 53828-54223 | OLP00333.1 | ATGCCCAGCACGCTCATTGAGGTCAGGCGCCAGTACACCGAAGCTGAAGAGGTGGCGATCATCGACGCGGTGCACGATGCGCTCGTGGCCGCGTTCCAGATACCCTCCGGTGACAAGCATGTCCGGCTGGTATCCCATGAACCACATCGGTTCTCGCATTCACCCGGACTGGCTCACCCCGAGCTGTACGCACTTGTCGCGATCGACTGCTTTGCCGGCCGATCGGTCGAGGCCAAGCGAAACCTCTACCGGGAGATCGTCACTCGCCTAGAGACACTGGGTATCCCGGCCGACCATGTCACGATCGTCCTGCGTGAGAGCGCGCTCGAGAACTGGGGCGTTCGTGGCGGGCGGGCCGCATGCGATGTCGATTTGGGTTTCGACGTCAACGTCTGA |
| *Mycobacterium salmoniphilum* DSM 43276 | MAFR01000001.1 | 64313-64669 | WP_078330277.1 | GGCCTGACCGCCACGGATTCCCCAGTTCTCTGTCACGCTCTCGCGCAGCACAATGGTGACGTGATCCGGCGGTATGCCCAGTAAGGAAAGCTGTTTCACAATCTTGGCATAGAGGTTTCGTTTCGCTCCTATCGATCGGCCCGCGAAGCAGTCGATGGAGACGAAGGTGTACAGCTCTGGCCGGGCCAGATTTGGCGCGTGCGAAAACCTATGGGGTTCATGCACAACAAGGCGCACATGCTTGTCCTCTTCCGGTATCTGAAAGGCAGTGACGAGCGCACCGTGAACCGCATCGATGATCGCCACCTCTTCGGCCTCGGTGTAGACCCGGCGGACCTCGACCACTGAACTGGGCAT |
| *Mycobacterium salmoniphilum* DSM 43276 (2) | MAFR01000030.1 | 87811-88197 | WP_078327763.1 | ATGCCGCTAGTGCGTATCGACGTCACCTCGGATCGGACGCGCGAACAGCAGCGCGCCATCGCCGACGCGGTGCACGAGGCCCTGGTCGAAGTCTTGAAAATCCCTGTGCGCGACCGATTCCAGATCATTACGGGGCATGACTCGGCGGATATCATCGCCGAAGATGCGGGACTGGGATTCCACCGATCGGCGCAGGTGGTGATCGTGCACATCTTCACCCAAGCCGGCCGGACGACGGAGACCAAACAGAAGGTCTTCTCGGCGCTGGCCGTGAAGCTGGCCGCCGTTGGCGTGGCGGGCGCGGATCTGTTCGTGGCGATCAGCGAAAACGGGTCGCAGGATTGGTCTTTCGGCTTCGGGCAGGCGCAATACGTCACCGGCGAGCTG |
| *Mycobacterium saopaulense* CCUG 66554 | MVII01000049.1 | 18416-18811 | ORB48254.1 | ATGCCACTAGTGCGTATCGACCTCACCTCGGACCGGTCCCGTGCAGAGCGGCGCGCCATCGCCGATGCCGTGCATGGCGGGCTGGTCGCGGTTCTGAAGATTCCCGAGCGAGACCGCTTCCAGATCATTACCGCACACGACCCGGCCGACATCATCGCCGAGGATGCGGGACTGGGATTCACGCGCTCGCAGGTGGTGATCGTGCACATCTTCACCCAGGGCGGCCGCACGGCGCAGACCAAGCAGCGGGTCTTCTCCGAGCTGGCGAACAGACTTGAGGCGGTCGGCGTCGCGGGAGCGGACTTATTCGTGGCAGTTAGCGAAAACGGGCCGCAGGATTGGTCTTTCGGCTTTGGCAAGGCACAGTACGTCACCGGCGAGTTGGCGGTGCCTGCC |
| *Mycobacterium saskatchewanense* DSM 44616 | LQPR01000049.1 | 70007-70393 | ORW68986.1 | CAACTCACCGGTGAGGAACTGCGCCCGGCCTTCGCCGAAAGACCAATCCTCGTCGCCGTTTTCGGTGATCGAGACGATCAGGTCGGCCGAGTCGAGGCCGCAGCGGTCGGCGAGGTTGGCCGCGAGCAGCTTGAAGAACTGCTCCTTCATGACGCGGGTGCGGCGCCGGCTCACCACGTGGACGATCACCAGCCGCGGTGAGCGGGTGAGCCCCAGACCGGTGTCCAGCGCGGCGATCTCGTGCGCCGGGTGGGTGCGCACCACCTGGTAGCGGTCGCGCGGCGGGACCCTGAACGCCTCGACGACGGCGTCGTGAATCGCGTCCAGCAGCTCCTGGACCTCCGACGGTGTTCGGCCCTCGATGAGGTCGACGTACAAAAGCGGCAT |
| *Mycobacterium scrofulaceum* DSM 43992 | MVIJ01000079.1 | 3348-3749 | ORB67392.1 | TGCCGAGGGTCATTGCAGCTCCCCGGTGAGGAATTGCGCCTGGCCGTGGCCGAAAGACCAGTCCTCGTCACCGTTTTCGGTGATCGTGACGATCAGGTCGGCCGGATCGACCCCGCACCGGTGGGCCAGGTTGGCGGCCAGCAACTCGTAGAACTTCTCTTTCATCGCCCGCGTGCGCCGGCGGCTCACCACGTGCACGATCACCTGTTGCGCCGTACGGGTGATGCCCAAACCGGTGTCCAGAGCGACGATCTCGTGGGCGGGATGGGTGCGCACTACCTGGTAGCGATCCCGCGCCGGCACCCCGAACGCTTCGACGACGGAGTGGTGAACCGCGTCGAGCAACCCCCGGACGTCGTCGGGGGCGCGGCCCTCGATCAGGTCGATGTACAGCAGCGGCAT |
| *Mycobacterium senegalense* DSM 43656 | JACKUT010000029.1 | 24669-25058 | MCV7337419.1 | TAGCAGACCCTCGATGAACTGCGCGCGCCCGTCACCAAAAGACCAGTCCGCGCGACTGTTCTCTACCATCGACACAATCAGATCCGCAGGTTTCAGGCCTGTTTGTTCGCGGAGCCGTTGCGCCAGAGCTCGGTACGTTTCTTGTTTCTGATCAGATGTGCGACCTTGCTGGAAGACGTGCACGAGGACAATGTCGTCGGTCCGGTCGAATCCGAGCCCTGTGTCTTCGGCGATGATCTGGCCCGGTTTGTGCTCGGTGATGATCTGGTACCTGTCGCGTTCAGGAGCGGCGAAATACTCAAGCATGACGTCTTGGATTGTGTCGGCGACCTGACGCAGCTGAGCCGGTGTGCGCCGGCCCTCGATGACATGAATATGTACGAGTGGCAT |
| *Mycobacterium senegalense* DSM 43656 (2) | JACKUT010000035.1 | 44040-44396 | MCV7338133.1 | ATGCCCAGCACGCTCATCGAGGTCAGGCGCCACTACACCGAATCCGAAGAGGTGGCGATAATCGACGCGGTGCACGATGCGCTCGTGGCGGCGTTTCAGATACCCGTCGGCGACAAGCATGTCCGACTGGTTTCCCATCAGCCACATCGGTTCTCGTACTCGCCGGGGTTGGCTCATCCCGAGCTCTACACGCTCGTCGGAATCGACTGCTTTTCCGGTCGATCAGTAGAGGCGAAGCGAAACCTCTACCGGCAGATCGTCATTCGGCTGGAGAAACTGGGCATTCCTGCCAACCATGTCACGATCGTCTTACGTGAGAGCCCGCTCGAGAACTGGGGCATCCGGGGCGGGCAGGCC |
| *Mycobacterium senriense* TY59 | AP024828.1 | 4468897-4469283 | BCZ24352.1 | ATGCCGCTGTTGTATGTCGACCTCGTCGAGGGCCGTTCGCCGTCGGAGGTTCGCGCGCTGCTGGACGCGATCCACGAGACGGTGGTTGAAGCGTTCGGTGTCCCGGAGCGGGATCGCTACCAGGTGGTGCACACCCACCCGGCACACGAGATCGTCACGCTGGATACCGGTCTCGGCATCGATCGTTCGTCTCGTCAGGTGGTCTTGCACGTGGTGAGCCGGCGACGTCCGCGCGAGCTCAAGCAGAAGTTCTATGAGTTGCTTGCGTCCCGTCTCGCGGACCGCTGCGGGCTCGACCCTGCCGACCTGATCGTCTCGGTCACCGAAAATGACGACGAGGACTGGTCTTTCGGCCACGGTAGGGCGCAGTTCCTCACCGGTGAGCTC |
| *Mycobacterium seoulense* DSM 44998 | JACKVP010000005.1 | 322555-322941 | MCV7436050.1 | CAGCTCCCCGGTGAGAAACTGTGCCCGGCCGTGGCCGAAAGACCAGTCCTCGTCACCGTTTTCGGTGACCGAGACGATCAGGTCGGCCGGGTCGAGCCCGCACCGCCCGGCGAGGTTGCCGGCCAGCAGTTCGTAGAACTTCTCCTTCATCGCCCGCGTGCGCCGCCGGCTCACCACGTGCACGATCACCTGGTGCGTCGACCGGGTGATGCCGAGGCCGGTGTCCAAAGCGACGATTTCGTGGGCGGGATGGGTGCGCACCACCTGATAGCGGTCACGCGGGGGCACCCCGAAGGCTTCGACGACGGACTCGTGAACCGCGTCGAGCAGCGCGCCGACCTCCGACGGCGTGCGGCCCTCGATGAGGTCGATGTATAGCAAGGGCAT |
| *Mycobacterium septicum* DSM 44393 | CBMO010000093.1 | 68203-68559 | WP_044519550.1 | GGCCTGCCCGCCACGAACGCCCCAGTTCTCCAGCGCGCTCTCCCGCAGCAGGATCGTGATGTGGCCGGAGGGGATGCCGAGATCCTCCAGACGAGTGACGATCTCCCGGTAGAGATTTCGTTTGGCCGGTACCGATCGACCGGCAAAGCAGTCGATGGTCACAAGCGTGTACAGCTCGGGGTTTGTCAGGCCCGGCGAATACGAGAACCGATGTGGTTCATGGGATAACAACCGGACATGCTTGTCGCCGGGAGGTATCTGGAATGCGGCCACCAGCGCATCGTGCACCGCGTCGATGATCGCGACCTCTTCTGATTCGGTGTACCGTCGCCTGACCTCGATGAGCGTGCTTGGCAT |
| *Mycobacterium sherrisii* ATCC BAA-832 | LQPT01000002.1 | 68604-68990 | ORW87419.1 | GTGCCGCTCATCTACATCGACCTCATCGAGGGACGCACGCCCACCGAGGTTCGCGACCTACAAGACGCTGTCCACGACGCCGTGGTTGAGGCGTTCGGCGTGCCCCCACGCGATCGCTACCAGGTCGTGCGCACTCATTCGAGGCACGAGATGGTGGCCTGGGACACCGGTCTGGGCATCGACCGCACGGAAAAGCTGGCCGTGGTGCATGTGGTGAGCAGACAGCGCACCCGGGCGATGAAGGAAAAGTTCTACGAGCTGCTGGCCACGAATCTGGCCGAGAACTGCGGGCTGGACCCGGCCGATCTGATCGTGTCGATCACCGAAAACGGCGACGAGGACTGGTCTTTCGGCCACGGCCGGGCGCAGTTTTTGACCGGGGAACTG |
| *Mycobacterium shigaense* UN-152 | MWON01000026.1 | 133254-133658 | PRI13719.1 | TGAGCCGGGTACTCATGCGAGCTCTCCGGTGAGAAATTGTGCCCGGCCATGGCCGAAAGACCAGTCCTCGTCTCCATTTTCGGTGATCGAGACAATCAGGTCGGCCGGTTCCACCCCGCATCGCTCGGCGAGGTTGGCTGCCAACAATTCGTAGAACCGTTCCTTCATGGCCCGGGTACGGCGCCGGCTCACCACGTGCACCACCACCAGCCGCGACGAGCGGTCGATACCCAAACCTGTGTCCCACGCGACGATTTCGTGCTCTGGATGGGTGTGCACCACTTGATAGCGGTCGCCCGGTGGCACGGCGAACGCTCCGACGACGGCGTCATGCGCCGCGTCCAGCAGCGCCCGGACCTCCGACGGGCTACGGCCCTCGATGAGATTGATGTACAGCAGCGGCAT |
| *Mycobacterium simiae* JCM 12377 | AP022568.1 | 5401485-5401871 | BBX43605.1 | ATGCCGCTGCTGTACCTCGACCAGATCGACGGTCGCAGCCCGTCGGAGATCCAGGCGTTGCTGGACGCCGCGCATGACGCCGTCGTCGACGCATTTGAGGTGCCGCCGGGTGACCGCTACCAGGTGGTGCGCACTCATCCAGCGCACGAGATTGTCGCCTGGGACACCGGTCTCGGCATCGACCGCTCGACGCGGCTGGTGATTGTGCATGTGGTCAGCCGCCGGCGCACCCGCGCGATGAAGGAACGATTCTACGAGTTGCTGGCGGCCAACCTCGCCGAGCGATGCGGGGTCGACCCGGCCGATCTGATCGTGTCGATCGCCGAAAATACAGACGCGGATTGGTCTTTCGGTCACGGTCGGGCGCAATTCCTCACTGGAGAACTG |
| *Mycobacterium simiae* JCM 12377 (2) | AP022568.1 | 2574580-2574960 | BBX40959.1 | TGTCGCGTACTGAGCTTCACCGTTGCCAAAGGACCAGTTATCTTTGGTGTTCTCCACCAGATTGATAACGATGTCCTCGCGTCGCATCCCGAGACGCTCGTGCAAGCCGTCCGCGATGGCTTTGTAAAACGCTGACTTCTTGTCCACGGTTCTGCCGGCCAGCAGCGTCACCTGGATGAACACGCAGTCATTCGACCGGCTGATGCCGAGGTAGTGGGGGTCAAAGATGAAGTTCTGCGCGCGATGTTCACTGATTACCTGGAAGCGATCCCCTTCAGGCACGCCGAGTGTGTCGACCATCGCGTTGTAGACGACATCGCCGATCGTTTGGCGATACTCGTCGGATTGCCCTTCTACGAGGTCGATTCGAGCGAATGGCAT |
| *Mycobacterium stephanolepidis* | AP018165.1 | 151537-151923 | BAX95509.1 | ATGCCATTAGTGCGTATCGACGTCACCTCGGATCGATCAGGCGATCAGCGACGCGCCATCGCCGACGCCGTGCACGAAGCCCTGGTCGAAGTCTTGAAAATCCCTGCACGAGACCGATTTCAGATCATCACTGCGCATGAGTCCGCCGACATCATCGCCGAGGATGCGGGGCTGGGGTTCGTGCGATCGGCCCGTGTGGTGATTGTGCACATCTTTACCCAGGCAGGCCGTACCACGGAGACCAAGCAGCGGATCTTCGCCGAGCTGGCTAAGAGGCTAGCCGCCGTCGATGTGGCGGGCGGGGACCTTTTCGTGGCGATCAGTGAAAATGGGTGGTCTTTCGGCTTCGGTAACGCGCAGTACATCACCGGTGAGCTGCCTGTGCCC |
| *Mycobacterium stephanolepidis* (2) | AP018165.1 | 3811796-3812152 | BAX99145.1 | CGCCTGACCTCCACGGATTCCCCAGTTCTCGGTCGCACTCTCGCGCAGCACAATGGTGACGTGATCCGGCGGTATCCCCAGTAAGGAAAGCCGTTTCACAATCTCGGCATAGAGATTACGTTTCGCCCCTATCGATCGGCCCGCGAAGCAGTCGATGGAAACGAAGGTGTACAGCTCCGGCCGGGCCAGGCTTGGCGCGTGCGAAAACCTGTGGGGTTCATGCACGACGAGGCGCACATGCTTGTCCCCTTCCGGTATCTGAAAGGCAGCGACCAGCGCGCCGTGGACCGCATCGATGATCGCCACCTCGTCGGCCTCGGTGTATCGCCGCCGGACCTCGACCACTGAACTGGGCAT |
| *Mycobacterium stomatepiae* DSM 45059 | JACKSO010000070.1 | 134711-135097 | MCV7165300.1 | CAGTTCTCCGGTGAGGAACTGCGCGCGACCGTGACCGAAAGACCAATCCTCGTCCCCGTTTTCGGTGATCGACACGATCAGATCGGCCGGGTCGACGCCGCATCGCTCGGCGAGGTTGGCCGCCAACAATTCGTAGAACCGTTCCTTCATGGCGCGAGTGCGCTGGCGACTGACCACGTGCACAATCACCAGCCGTGCCGAGCGGTCGATGCCGAGACCGGTGTCCCAGGCCACGATCTCGTGCGCGGGATGAGTGCGCACCACCTGGTAGCGGTCGCCCGGCGGGACCGCGAACGCGCCGAGGACGGCGTCATGCGCGGCGTCCAGCAACGCCTGGATCTCCGACGGCGTGCGACCCTCGATCAGGTCGAAGTACAGCAGCGGCAT |
| *Mycobacterium syngnathidarum* 27335 | MLCL01000054.1 | 35978-36412 | OLT95885.1 | CCCGGCCTCGGGCGGCACGAGAGGTCCCGTTGACACCGATCAGACGTTGACCGCGAAGCCAAGATCGACGTCGCACGCGGCCTGTCCGCCTCGTACGCCCCAGTTCTCGAGTGCGCTCTCACGCAAGACAATCGTGACGTGGTTTGCAGGGATGCCCAGCGCCTGCAGACGAGTGGTGATCTCCCGGTAGAGATTCCGCTTGGCCTCGACCGATCGACCGGCGAAGCAGTCGATCGCAACGAACGTGTACAGCTCGGGGTGAGCCAATCCGGGGGAATACGAGAACCGATGCGGTTCATGGGATACCAATCGGACATGCTTGTCGCCGGGAGGTATCTGGAACGCGGACACCAGCGCATCGTGCACCGCATCGATGATCGCCACCTCGTCGGATTCGGTGTACGGGCGCCTGACCTCGATGAGCGTGCTGGGCAT |
| *Mycobacterium talmoniae* ATCC BAA-2683 | PPEA01000842.1 | 167-553 | PQM44343.1 | GAGTTCTCCGGTGAGAAACTGGGCCCGACCGTGGCCGAACGACCAGTCCTCATCGCCGTTTTCGGTGACCGAAACGATCAGATCGGCGGGGTCCAGGCCGCACCGGTCGGCAAGCTTGCCGGCCAGCAGGTCATAGAACCGCTGCTTCTGCGCGCGGGGGCGTCGGCGGCTGATCATGTGCACGATCACCAGCCGCGCCGAGCGGGTGATACCCAGGCCGGTGTCCAACGCGACGACCTCGTCGGCCCGGTGCGTGCGGACCACCTGGTAGCGGTCCCGCGGGGGCACCGCGAACGCCTCCAGGACGGCCTCATGGATGGTGTCCAGCACGGCTTGGACCTCGGCGGGTGTGCGGCCCTCAATGAGATCGACGTAGATCAGCGGCAT |
| *Mycobacterium talmoniae* ATCC BAA-2683 (2) | PPEA01000802.1 | 478-834 | PQM44444.1 | ATGCCCAGCTCACTGATCGAGGTTCGCCGCCACTACACGCCCGCCGAGGAAGTCGCCCTCATCGATGCGGTCCACGCCGCGCTGGTGGCCGCGTTCCAGATCCCGGCCGCGGACAAGAATGTGCGGCTGGTCGCCCACGAGCCGCATCGGTTCGCCCACTCGCCGAACCTGACGCACCCGGAGCGCTACACCCTGGTGACCATCGACTGTTTCGCCGGCCGATCCGTGGACGCCAAACGGAACCTCTACGGCGAGATCGTGCGTCGGCTCGCCGAACTCGGTATCCCCGCCGACCACGTCCGGATCCTGCTGCGCGAGAGCGCGCCGGAGAATTGGGGCATCCGCGGCGGGCGCGCC |
| *Mycobacterium terrae* CIP 104321 | LQPX01000014.1 | 100394-100750 | ORW94928.1 | ATGCCCACTGTCCTGATCGAGGTCCGTCGCCGCTACGAGCCGGCCGAAGAAGTCGCGATCATCGACGCCGTCCACGGCGCGCTGGTGACCGCCTTCCAGATCCCGGCCCAGGACAAGAACGTGCGGCTGATCGCCCATGAACCGCACCGGTTCGCGGTTCCCGAGCACCTGGCGCAGCCCGAGTACCGCACCCTGATCTCGATCGACTGCTTCACCGGCCGGTCCCTGGACGCCAAGCGGGCCCTCTACGCCGAGATCGTGGAACGGCTTGCCGCGTTGGGTATTCCGCGTGACCATGTGATGATCACGCTGCACGAGGTCGACCGGCAGAACTGGGGAATCCGCGGCGGCCAGGCC |
| *Mycobacterium terramassiliense* AB308 | FTRV01000008.1 | 260082-260489 | SPM26798.1 | ATGCCGTTGTTGTACATCGACCTCATCGAGGGCCGCACGCCGGCGCAGGTTCGGGCGCTGCTGGACGCCGTCCACGAGGCGGTGGTCGAGGCGTTCGGCGTGCCGCCGCGCGACCGCTACCAGGTGGTGCGCACCCATCCGGCCCACGAAATCGTCGCCTGGGACACCGGCCTTGGCATCACCCGGTCGGCGCAGCAGGTGATCGTGCACATGGTGAGCCGGCGACGCACCCGGGCGTTGAAGGAGAAGTTCTACGAGCTGCTGGCGCTCAGCCTCTCCGAGAGGTGCGGGATCGACCCTGCCGATCTGATCGTCTCGATCACCGAAAACGGCGATGAGGATTGGTCTTTCGGGCACGGCCGCGCGCAGTTCCTTACCGGGGAGCTGACATGACCCTGGACACCCGCC |
| *Mycobacterium timonense* CCUG 56329 | JACKSY010000048.1 | 96257-96646 | MCV6995777.1 | ATGCCGCTGTTGTATATCGACCTCATCGAGGGTCGGACGCCCTCGGAGGTCAGTGCCCTGCTGGACGCGATCCACGACACGGTCGTCGAGGCGTTCGGCGTTCCGCCGCGGGATCGCTACCAGGTGGTGCATACCCACCCGGCACACGAAATCGTTACGCTGGACACCGGTCTGGGCATCGATCGGTCGTCTCGCCAGGTGGTCTTGCACGTGGTGAGCCGACGGCGGCCGCGTGAGCTCAAGCAGAAGTTCTATGAGCTGCTGGCGTCGCGCCTCGCCGGCCGGTGCGGGCTCGACCCCGCCGACCTGATCGTCTCGATCACCGAAAACGACGACGAGGACTGGTCTTTCGGCCACGGGAGGGCTCAGTTCCTGACCGGTGAACTCACG |
| *Mycobacterium trivialis* DSM 44153 | LQPZ01000013.1 | 161162-161554 | ORX07000.1 | ATGCCCAACTCCGTGATCGAGATCCGCCGGACTTACTCCCGCGACGAGGAAACGGCGCTGATCGAGGCGGTGCACGCCGCGCTGATGGCCGCGTTCGACGTGCCCGCCGACGGCCGCTGCGTGCGACTACTGGTCCACGAACCGCACCGGTTCGCCTGCCCGGCGAAGCTCGCCGACCCCGAGCGGTACACGCTGGTCACTATCGACTGCTTCGCCGGCCGCTCACTGCAGGCCAAACGCGCGCTCTACCGCGGCATCGTCGAACGTCTCGCCGCGTTGGGCATCCCGGGTGACCACGTCGCGATCGTGGTCCGGGAGAACACCAAGGAGAACTTCGGGATTCGCGGCGGGCGGGCGGCCTGCGACGTCGACCTGGGCTACACCGTCACGGTG |
| *Mycobacterium virginiense* DSM100883 | CP092430.2 | 1663238-1663594 | ULP49012.1 | ATGCCCACCGTCCTGATCGAGGTCCGTCGCCACTACGAGCCGGCCGAGGAAGTCGCGATCATCGATGCCGTCCATGGCGCGCTCGTGACCGCTTTCCAGATCCCGGCCAAGGACAAGAACGTGCGGCTGGTGGTGCACGAGCCGCACCGTTTTGCGGTTCCGGCGCAGCTCGAGAAGCCCGAATGCCGCACCCTGATCTCGATCGACTGCTTCTCCGGCCGGTCGCTGGAGGCCAAGCGCCTGCTTTATGCCGGGATCGTCGAAAATCTTGCCGCGCTGGGTATTCCCGCCGACCACGTGATGATCACCCTGCATGAGGTGGATCGGGAGAACTGGGGCATCCGGGGCGGGCAGGCC |
| *Mycobacterium vulneris* DSM 45247 | CCBG010000001.1 | 2581071-2581463 | CDO29684.1 | ATGCCCAGCACGCTCATCGAAGTCCGGCGCGAGTACACCGAAATCGAAGAAGTGGCGATCATCGATGCGGTGCATGATGCGCTCGTGGCCGCGTTCCAGATACCCCGCGGCGACAAGCATGTTCGGCTGGTGTCCCACGAACCTCATCGGTTCTCGTATTCACCCGGACTGGCTCGCCCCGAGCTCTACACACTTGTCGCGATCGACTGCTTTGCCGGCCGATCCATCGAGGCCAAGCGAAATCTCTACCGGGAGATCGTCACTCGCCTGGAGGTACTGGGCATCCCAGCCGACCATGTCACGATCGTCTTGCGTGAGAGTGCGCTCGAGAACTGGGGTGTCCGGGGCGGGCAGGCCGCGTGCGACATCGATCTGGGCTTCGACGTCAACGTC |
| *Mycobacterium wolinskyi* ATCC 700010 | LQQA01000007.1 | 57127-57522 | ORX17486.1 | ATGCCACTGGTTCGTATCGATCTGCTGAACCACCATGGAAATGAACACATCCGAAACGTCGCCGACGCCATCCACCGAAGCCTGGTCGCGGTACTCGGAATCCCCGAGCGTGACCGGTTCCAGGTCATCACCCCGCATCGCCCCGGAGAGATCATCGCGCTGGACGCCGGCCTCGGGTTCGACCGCTCGCCGGACACCGTGATCATCCAGATCTTCACGCAGCGCGGGCGTTCCATCGACACGAAGGAACGGCTCTACGCCGAGATCGCGGCGCAGCTGGAGGCGGTGGGAGTCCGCGGCGACGACATTTTCGTCGGCATCGCAGAGAACGGACCCGAGGACTGGTCGTTCGGTTTCGGAAAGGCCCAGTACGTCACCGGCGAACTCGCCGTACCG |

**Supplementary Table 3.** BLASTn analysis of MSAD-1 genes of 5 *Mycobacterium* strains subject to lateral gene transfer.

| Query sequence | strain | Query Cover | E value | Per. Ident | Accession |
| --- | --- | --- | --- | --- | --- |
| *M. agri* | *Aeromicrobium erythreum* strain AR18 | 97% | 8.00E-41 | 70.63% | CP011502.1 |
|  | *Kocuria rosea* strain ATCC 186 | 96% | 7.00E-54 | 72.96% | CP035103.1 |
|  | *Rhodococcus pseudokoreensis* strain R79 | 95% | 1.00E-69 | 75.72% | CP070619.1 |
|  | *Gordonia bronchialis* DSM 43247 | 95% | 3.00E-46 | 72.16% | CP001802.1 |
| *M. senegalense* | *Kocuria rosea* strain ATCC 186 | 99% | 2.00E-53 | 72.75% | CP035103.1 |
|  | *Rhodococcus pseudokoreensis* strain R79 | 98% | 3.00E-45 | 71.02% | CP070619.1 |
|  | *Kocuria flava* strain HO-9041 | 98% | 4.00E-44 | 70.76% | CP013254.1 |
| *M. farcinogenes* | *Kocuria rosea* strain ATCC 186 | 99% | 2.00E-53 | 72.75% | CP035103.1 |
|  | *Rhodococcus pseudokoreensis* strain R79 | 98% | 3.00E-45 | 71.02% | CP070619.1 |
|  | *Kocuria flava* strain HO-9041 | 98% | 4.00E-44 | 70.76% | CP013254.1 |
| *M. dioxanotrophicus* | *Kocuria turfanensis* strain HO-9042 | 99% | 3.00E-58 | 73.85% | CP014480.1 |
|  | *Rhodococcus pseudokoreensis* strain R79 | 96% | 1.00E-62 | 74.67% | CP070619.1 |
|  | *Kocuria rosea* strain ATCC 186 | 95% | 2.00E-60 | 74.67% | CP035103.1 |
|  | *Gordonia bronchialis* DSM 43247 | 95% | 6.00E-36 | 70.21% | CP001802.1 |
|  | *Brachybacterium saurashtrense* strain DSM 23186 | 90% | 9.00E-40 | 72.07% | CP031356.1 |
| *M. simiae*** | *Methylobacterium durans* strain 17SD2-17 | 98% | 3.00E-51 | 73.89% | CP029550.1 |
| Alignments were performed with Basic Local Alignment Search Tool (BLAST) program | | | | | |
