# Supplementary material for: Phylogenetic distribution of malonate semialdehyde decarboxylase (MSAD) genes among strains within the genus Mycobacterium: evidence of MSAD gene loss in the evolution of pathogenic mycobacteria
Source: Front Microbiol. 2023 Oct 13;14:1275616. doi: 10.3389/fmicb.2023.1275616 (PMC10606566; doi:10.3389/fmicb.2023.1275616)
Supplement: Supplementary file 2 [file Table_2.docx]

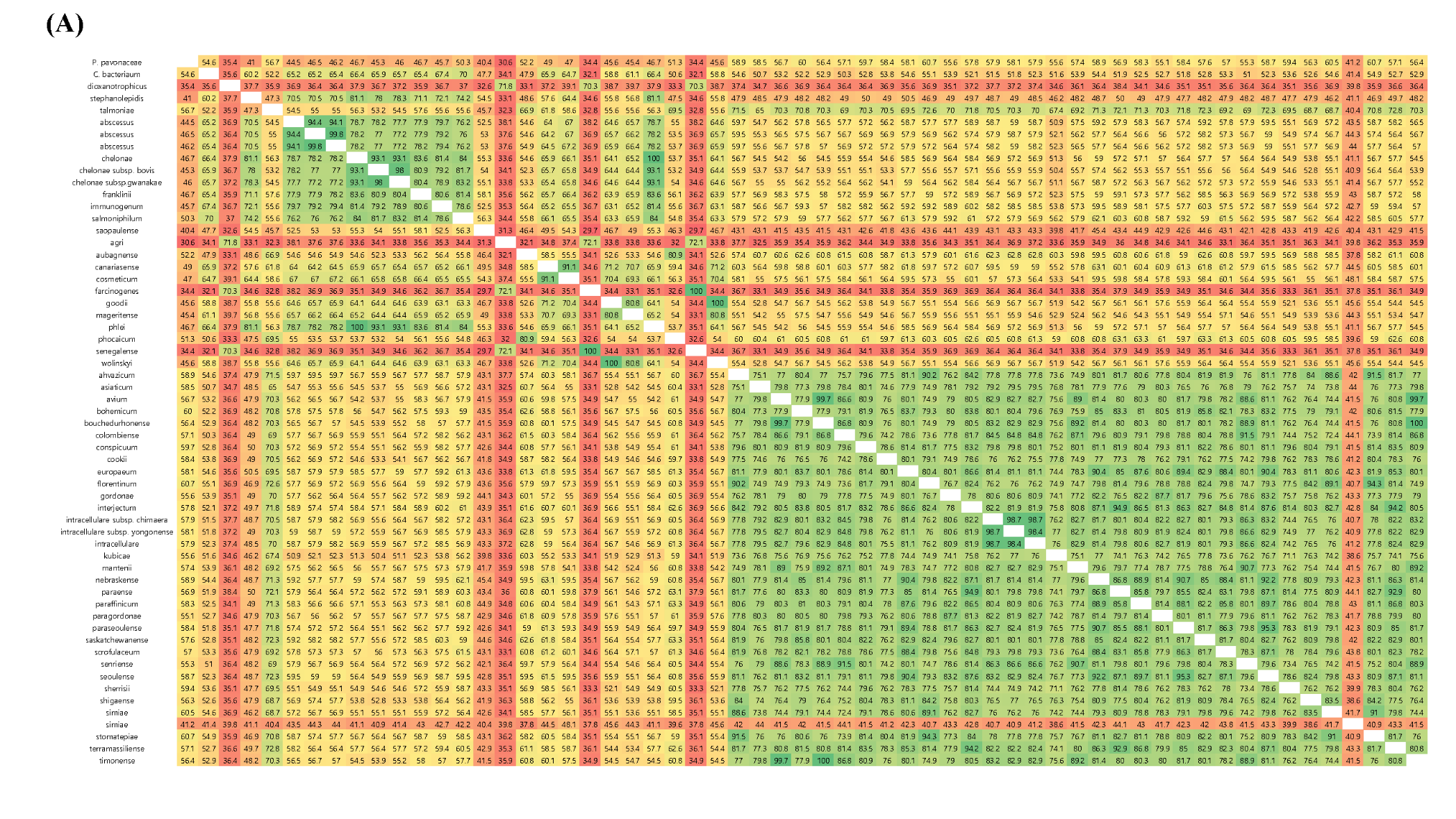


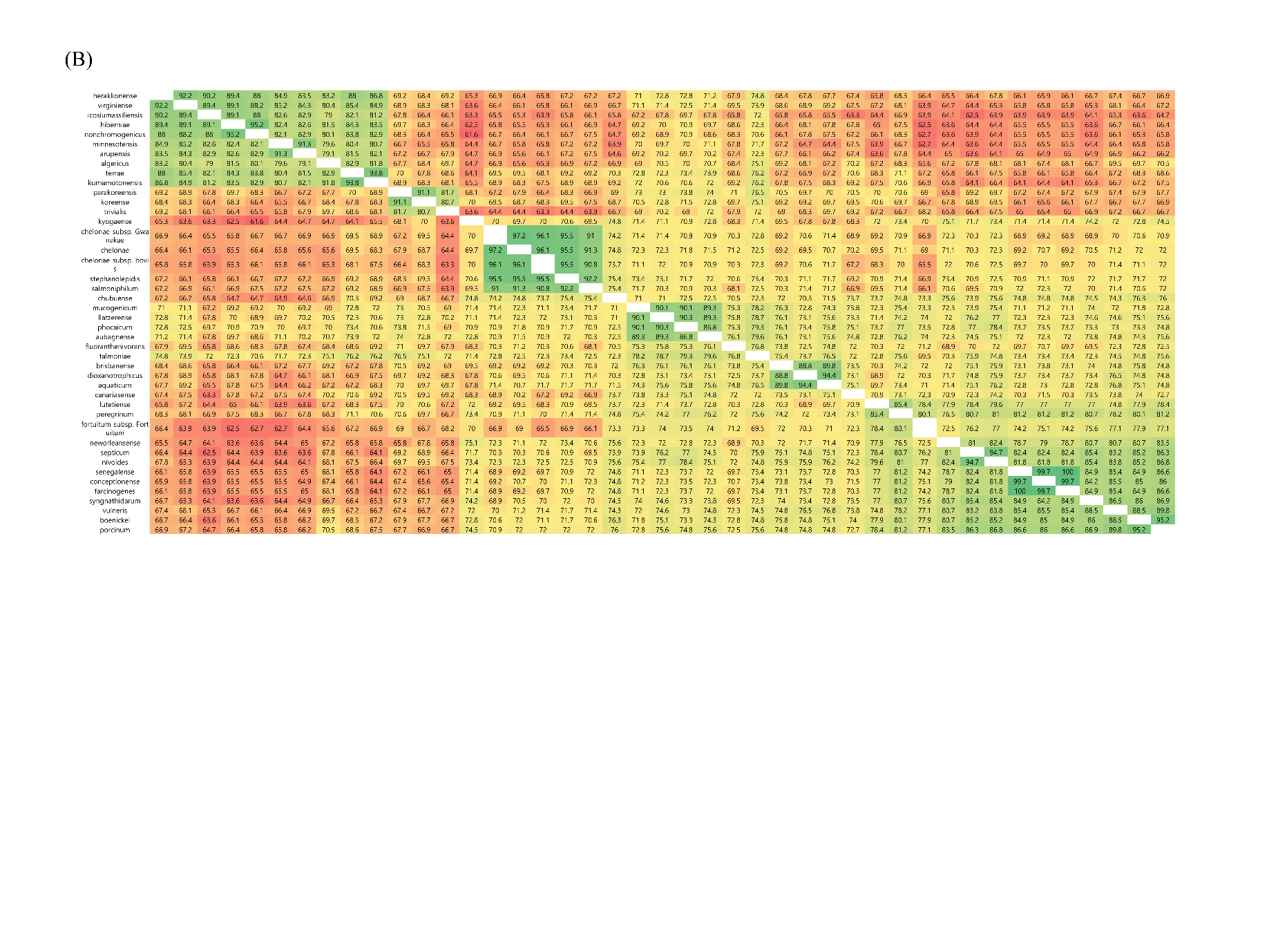


**Supplementary Figure 1.** These matrices show the average nucleotide identity. The numbers are a similarity index between two genomes expressed as a percentage. Nucleotide similarity analysis of **(A)** MSAD-1 and **(B)** MSAD-2.


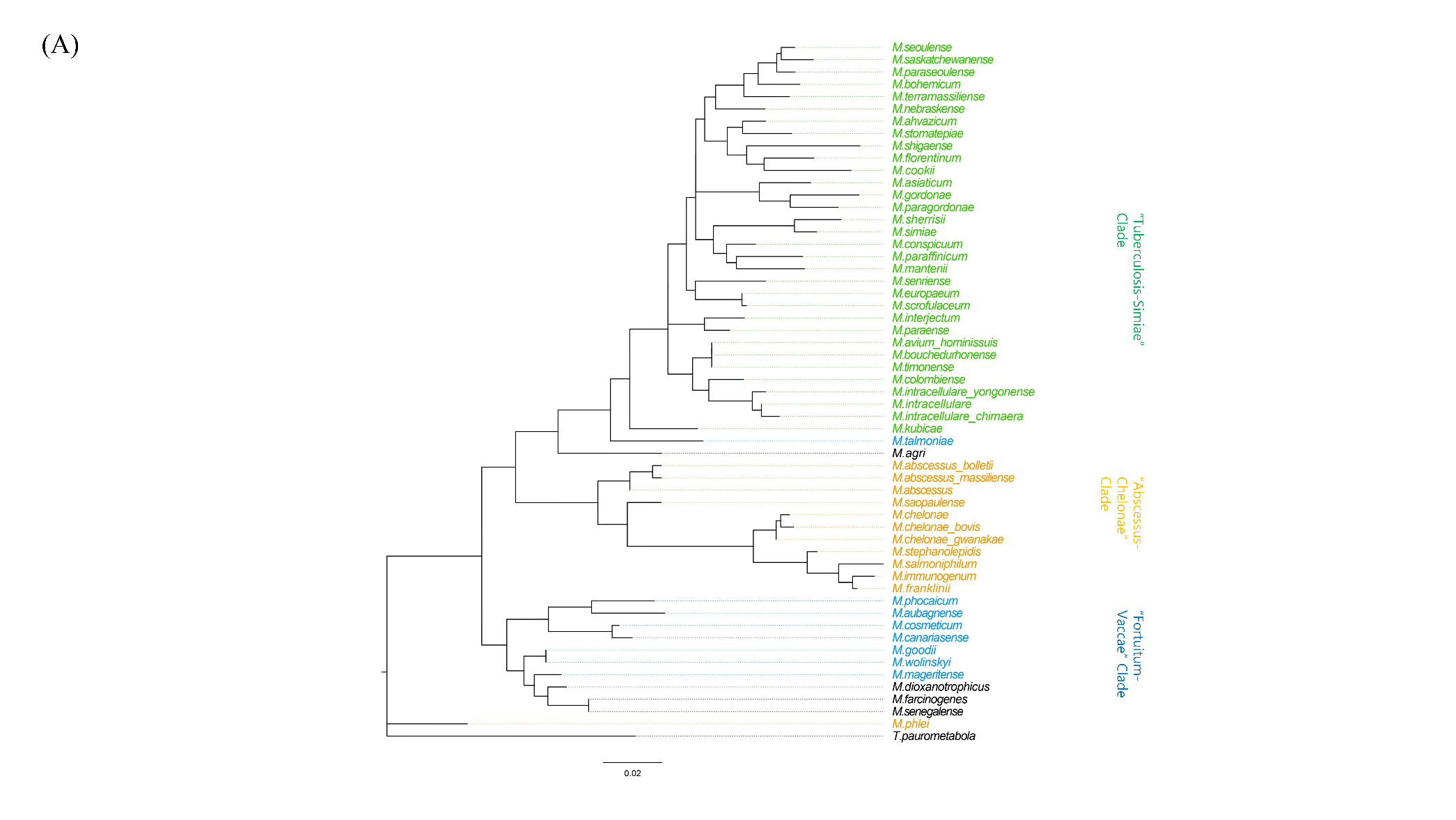


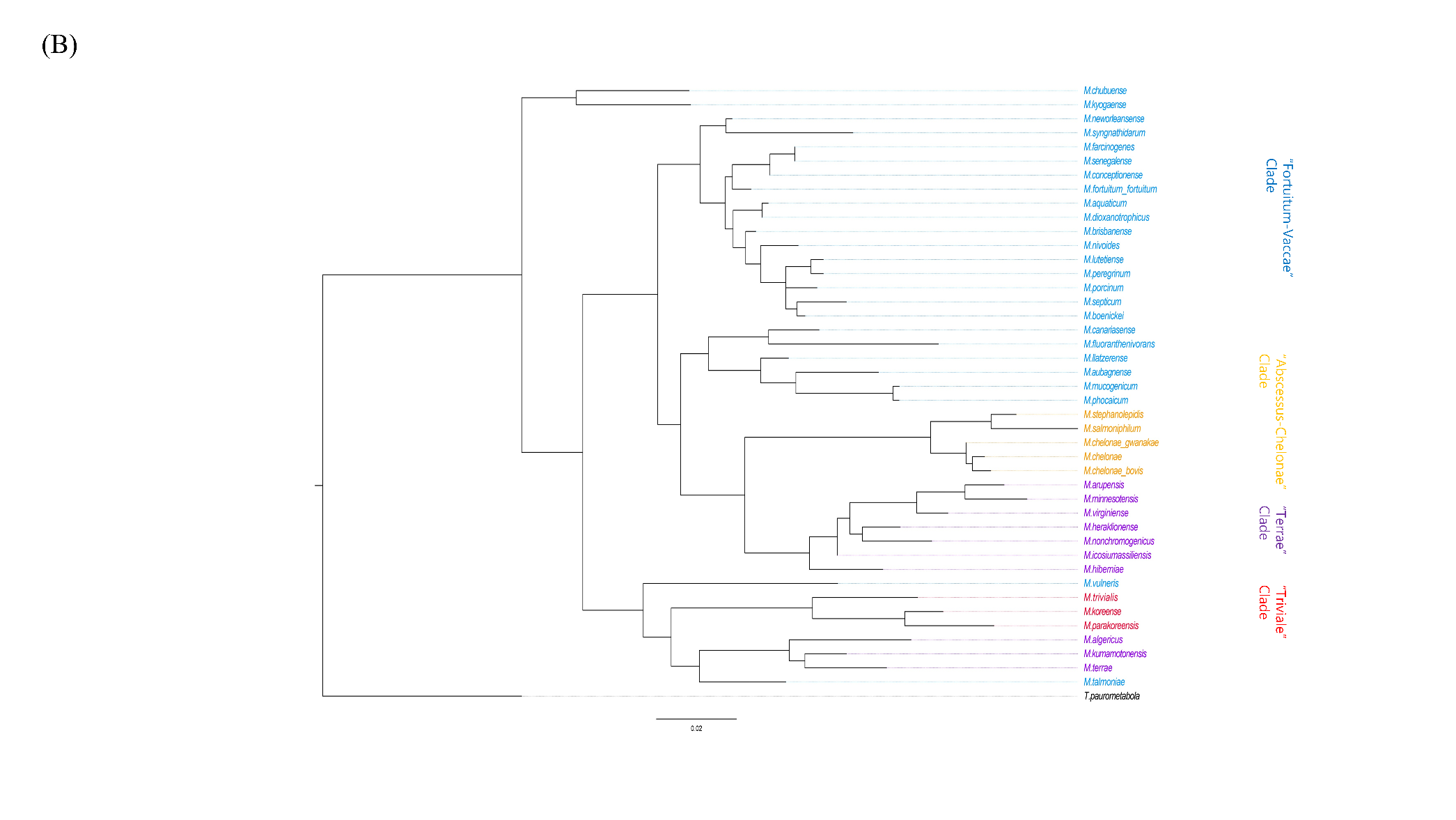


**Supplementary Figure 2.** The 644 bp based hsp65 gene-based phylogenetic trees from 56 selected mycobacterial strains, i.e., **(A)** the MASD-1 tree, and from 43 selected mycobacterial strains, i.e., **(B)** the MASD-2 tree.


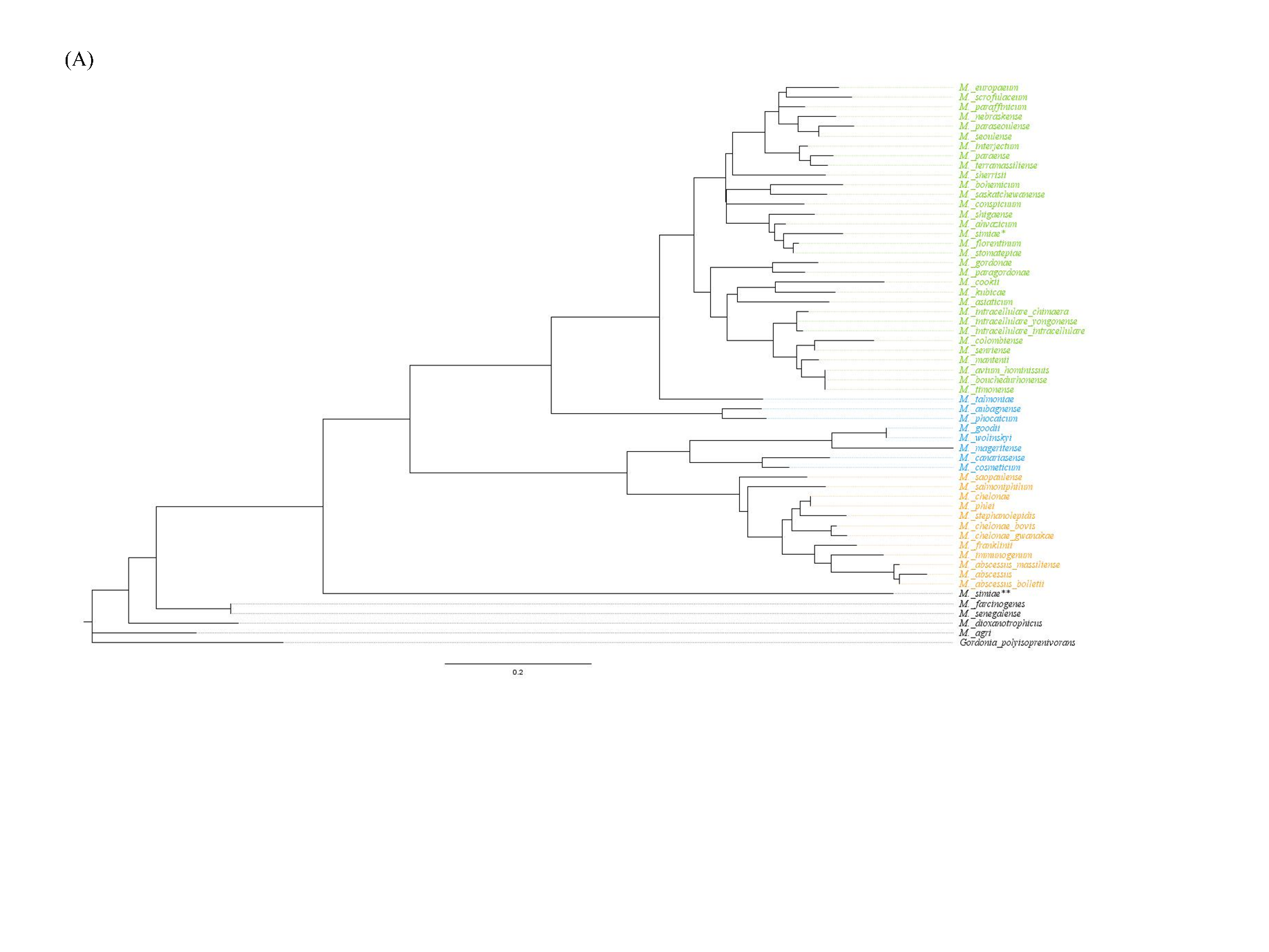


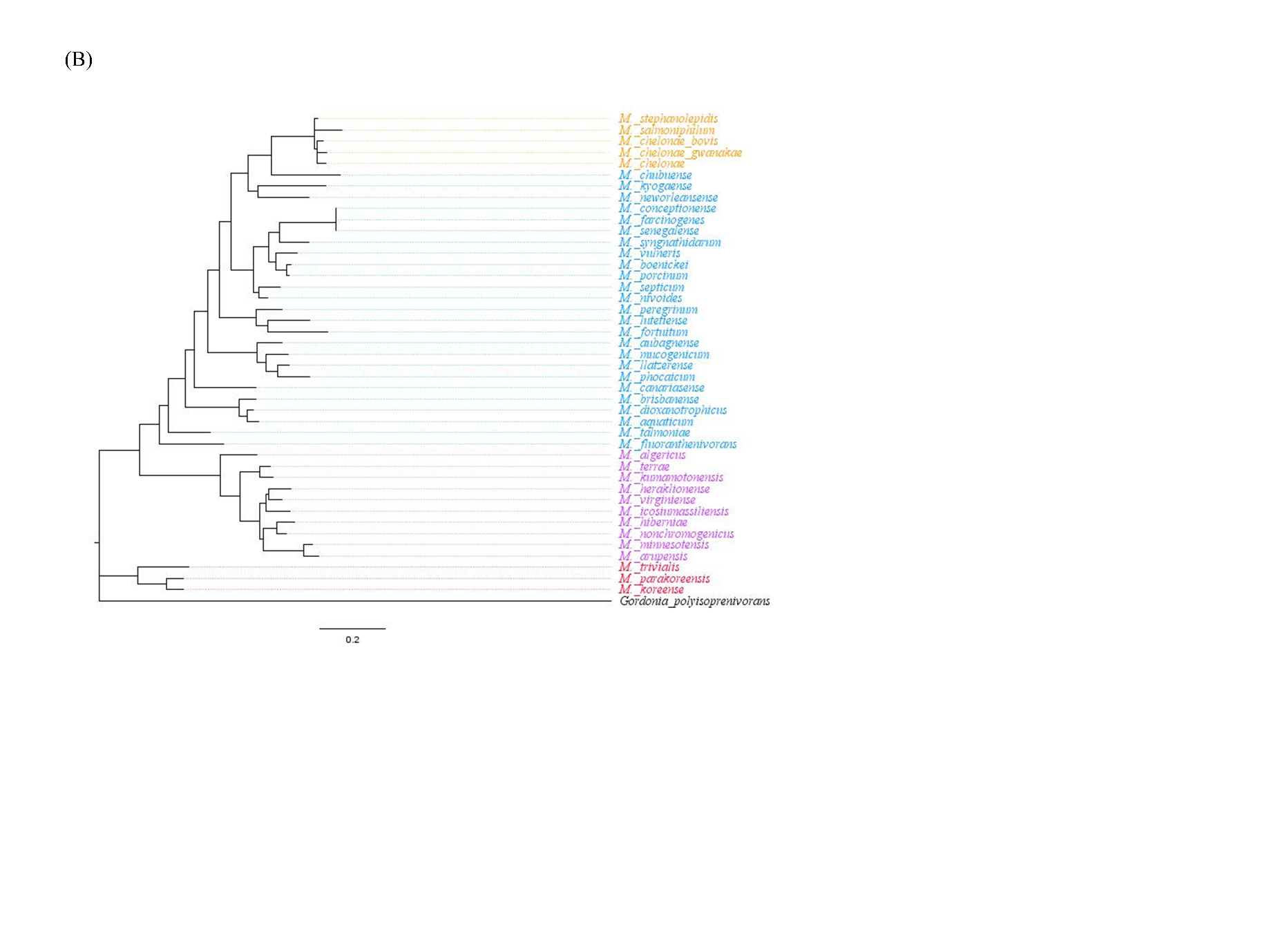


**Supplementary Figure 3**. Phylogenetic trees based on the MSAD-1 and MSAD-2 protein sequences. **(A)** The mycobacterial MSAD-1 phylogenetic tree was constructed from protein sequences of 57 mycobacterial MSAD-1s from 56 mycobacterial strains (two independent MSAD-1s in *M. simiae*). **(B)** The MSAD-2 phylogenetic analysis was constructed using 43 protein sequences of mycobacterial MSAD-2.
